# The best alternative for estimating reference crop evapotranspiration in different sub-regions of mainland China

**DOI:** 10.1038/s41598-017-05660-y

**Published:** 2017-07-14

**Authors:** Lingling Peng, Yi Li, Hao Feng

**Affiliations:** 1 College of Water Resources and Architecture Engineering, Northwest A&F University, Yangling, 712100 China; 2 Institute of Water-Saving Agriculture in Arid Areas of China, Northwest A&F University, Yangling, 712100 China; 3 Institute of Soil and Water Conservation, Chinese Academy of Sciences and Ministry of Water Resources, Yangling, 712100 China

## Abstract

Reference crop evapotranspiration (*ET*_o_) is a critically important parameter for climatological, hydrological and agricultural management. The FAO56 Penman-Monteith (PM) equation has been recommended as the standardized *ET*_o_ (*ET*_o,s_) equation, but it has a high requirements of climatic data. There is a practical need for finding a best alternative method to estimate *ET*_o_ in the regions where full climatic data are lacking. A comprehensive comparison for the spatiotemporal variations, relative errors, standard deviations and Nash-Sutcliffe efficacy coefficients of monthly or annual *ET*_o,s_ and *ET*_o,i_ (*i* = 1, 2, …, 10) values estimated by 10 selected methods (i.e., Irmak *et al*., Makkink, Priestley-Taylor, Hargreaves-Samani, Droogers-Allen, Berti *et al*., Doorenbos-Pruitt, Wright and Valiantzas, respectively) using data at 552 sites over 1961–2013 in mainland China. The method proposed by Berti *et al*. (2014) was selected as the best alternative of FAO56-PM because it was simple in computation process, only utilized temperature data, had generally good accuracy in describing spatiotemporal characteristics of *ET*_o,s_ in different sub-regions and mainland China, and correlated linearly to the FAO56-PM method very well. The parameters of the linear correlations between *ET*_o_ of the two methods are calibrated for each site with the smallest determination of coefficient being 0.87.

## Introduction

Atmospheric water demand has been described using potential evaporation (*ET*_p_), pan evaporation, and reference crop evapotranspiration (*ET*_o_). *ET*_p_ is the amount of water transpired in a given time by a short green crop, completely shading the ground, of uniform height and with adequate soil water status in the profile^1, 2^. *ET*_p_ has been applied as an important parameter for several decades in the field of hydrology, meteorology, agricultural engineering, etc. However, crop conditions in *ET*_p_ estimation was assumed constant. To avoid ambiguities involved in the definition and interpretation of *ET*_p_, *ET*_o_ was introduced by irrigation engineers and researchers in the late 1970s and early 1980s^3, 4^. *ET*_o_ is evapotranspiration rate from a hypothetical grass reference crop with a height of 0.12 m, a fixed surface resistance of 70 sec m^−1^ and an albedo of 0.23, actively growing, well-watered, and completely shading the ground^5^. *ET*_o_ incorporates multi-climatic factors and expresses the evaporative demand of the atmosphere independent of crop type, crop development and management practices. Under the world-widely accepted global warming background^6^, *ET*_o_ has become an important agrometeorological parameter for climatological and hydrological studies, as well as for irrigation planning and management^7–9^. *ET*_o_ has also been incorporated in drought severity and evolution analysis^10–12^. The application of *ET*_o_ was also related to water use of crops^13^. *ET*_o_ has been widely used in different research fields with various objectives because it can be computed from meteorological data.

The methods for estimating *ET*_o_ (*ET*_p_) could be classified as empirical, temperature-based, radiation-based, pan, and combination types. In recent years, several simplified *ET*_o_ equations were proposed and validated for their applicability^14^. Of these methods, the temperature-based equations, such as the Thornthwaite^1^, the Blaney-Criddle^15^, and the Hargreaves-Samani (1985a)^16, 17^, were extensively adopted because they mainly use easily-obtained tempereature data. The radiation-based methods were also applied^18^. The pan evaporation methods were used when the observed pan data were available^19–22^. The physically-based combination methods explicitly incorporate physiological and aerodynamic parameters^3, 5, 23^. The Penman-Monteith (PM) equation was selected as the standard *ET*_o_ estimation method by the Food and Agriculture Organization (FAO) of the United Nations because it closely approximates *ET*_o_ at the locations evaluated^5, 24, 25^. Afterwards the FAO56-PM equation was world widely applied for the validation of the other equations in absence of experimental measurements^26^.

In recent years, variations of FAO56-PM-based *ET*_o_ (*ET*_o,s_) has been extensively investigated since the FAO56-PM was recommended as a standard *ET*_o_ estimation method^27–31^. The *ET*_o,s_ variations were also analyzed in partial or entire mainland China (EMC) concerning different application objectives^32–43^. Meanwhile, the evaluation of the FAO56-PM method has also been widely conducted by comparing with different *ET*_o_ estimation methods. The evaluation research mainly focused on answering which method could be an alternative for FAO56-PM either the input data were full, limited or missing^9, 44–46^. It is known that the FAO56-PM equation requires a large data input for estimating *ET*_o_, including the geological variables such as elevation and latitude, and the meteorological variables such as minimum air temperature (*T*_min_), average air temperature (*T*_ave_), maximum temperature (*T*_max_), wind speed, relative humidity (*RH*) and sunshine hour (*n*). The high data demand of the FAO56-PM method realized its overall high accuracy, but restricted its application in some data-lacking regions. In the regions where the observed long-term meteorological data are difficult to obtain, the FAO56-PM method is not the best choice. To solve this problem, *ET*_o_ estimation methods with a lower data requirement and a simpler computation process are preferentially applied.

Although *ET*_p_ and *ET*_o_ are not equivalent terms, both provide estimates of atmospheric evaporative demand. In the previous studies, there are different understandings about the relationship between *ET*_p_ and *ET*_o_. Several researchers differ the two items strictly^32, 47, 48^. For instance, FAO56-PM *ET*_o_ equation is considered a PM *ET*_p_ equation for specific reference conditions^47^. For strictly utilization of the items, Allen *et al*.^5^ strongly discourage the use of *ET*_p_ for *ET*_o_ estimation concerned about the ambiguities in their definitions. A few researchers consider *ET*_o_ is a kind of *ET*_p_^49^ or it is reference values of *ET*_p_ for a uniform grass reference surface^50^. Noticeably, a lot of researchers look upon *ET*_p_ and *ET*_o_ as identical concepts and share similar equations for their estimations^51–55^. Usually, a climatologist or meteorologist and a hydrologist use the term “potential”, whereas an irrigation scientist uses the term “reference crop”, although the estimation equation could be same. Even some equation-proposers potentially identified the two items, such as Hargreaves and Samani^16, 17^ adopted “potential” while Hargreaves and Samani^16^ used the term “reference crop”. Ambiguity between *ET*_o_ and *ET*_p_ was expected to be reduced by more extensive definition of *ET*_p_ as potential crop evapotranspiration or by using one of the *ET*_o_ definitions^56^. Take Thornthwaite^1^ for another example, this method was originally proposed to estimate *ET*_p_, but was also applied for estimating *ET*_o_ in different cases^57, 58^. Therefore, although there are differences between *ET*_p_ and *ET*_o_, there is close relationship between them and their estimations could be quantitatively linked.

China has a total land area of 9,597,000 km^2^ and is the third largest country in the world. It has a complicated geomorphology which contains different water bodies, glaciers, frozen soils, deserts, basins, mountains, farmland, and forests. The elevations are general lower and lower from the west to the east, shaping a so called “3-level-catena” landform^59^. The weather stations in China distributed non-uniformly, there are more weather stations in the eastern China, but less in the western regions, especially less on Qinghai-Tibet Plateau. Neither is the distribution of the sites even, nor are the observed climatic elements same for different stations. The total sites available for air temperature and precipitation data are as large as 2474^60^, but when more climatic elements are needed, data from much less number of sites were available, estimated *ET*_o,s_ values for 200 sites in China by Fan *et al*.^35^, while 552 sites are suitable for *ET*_o,s_ analysis of this research. Not only in China, similar phenomena of difficulty in acquiring long-term and full weather data are also common in other developing countries because of some natural (geographical and climate) and humanity (economic power, knowledge and technology) reasons. Under this condition, for the *ET*_o_ estimation of China, to date to calibrate a suitable alternative equation which is simpler in computation process using less weather data and has a general good accuracy when compared to the FAO56-PM equation, are still very important for different sub-regions of China and EMC. Although performance of 16 different *ET*_o_ equations were compared for Xiaotangshan, Changping, Beijing in North China Plain^61^, a thorough and detail research for selecting a best alternative in EMC has not been conducted.

Based on the reasonable selection of 11 different *ET*_o_ estimation methods for the calculation of monthly *ET*_o_, this research aims to: (1) investigate the spatiotemporal variations and the trends of *ET*_o_ using climatic data from the selected 552 sites in EMC; (2) compare the performance of the 10 selected *ET*_o_ estimation methods with the standard FAO56-PM method in different sub-regions of China and EMC for the period 1961–2013; (3) select a best alternative of the FAO56-PM *ET*_o_ equation, which would be simpler in *ET*_o_ estimation and use less climatic variables; and (4) calibrate *ET*_o_ using the alternative equation with the standard FAO56-PM equation.

## Data and Methodology

### Data

**Figure 1:**
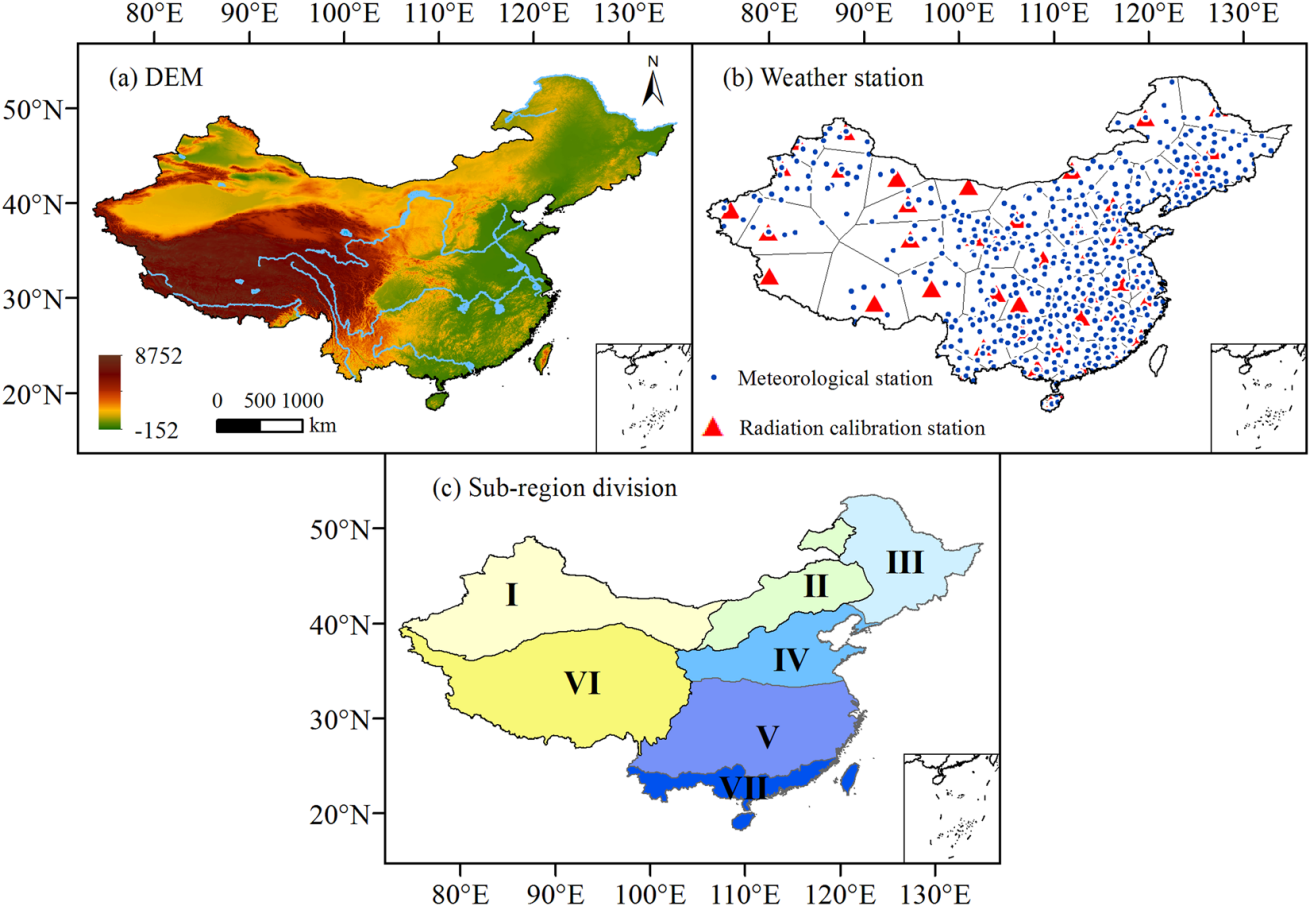
The DEM, weather station distribution and the sub-region division (I to VII) in China. (ArcGIS 10.2, http://map.baidu.com, Lingling Peng).

Geographical and weather data from 552 National Meteorological Observatory stations in EMC were collected from the China Meteorological Administration. The data contained both the daily and monthly timescales. The weather data included *T*_max_, *T*_min_, *T*_ave_, *U*_10_ wind speed at 10 m, *RH*, and *n*. The data duration was 1961–2013. The elevations of the selected sites covered a large range in EMC (Fig. 1a). To obtain more accurate *ET*_o_ estimation, the 48 sites reported by Chen *et al*.^62^ were used as the radiation correction station. Meteorological station (marked with blue circle) and radiation calibration station (marked with red triangle) and they were set as the centers of the Thiessen polygons to find the other sites which would use same parameters with them for estimating radiation (Fig. 1b). The EMC is divided into seven sub-regions^63^ considering the differences in topography and climate^64^ (Fig. 1c). Including the temperate and warm-temperate desert of Northwest China (sub-region I, 61 sites), the temperate grassland of Inner Mongolia (sub-region II, 44 sites), the warm-temperate humid and sub-humid Northeast China (sub-region III, 72 sites), the warm-temperate humid and sub-humid North China (sub-region IV, 104 sites), the subtropical humid Central and South China (sub-region V, 165 sites), the Qinghai-Tibetan Plateau(sub-region VI, 49 sites), and the tropical humid South China (sub-region VII, 57 sites).

### Estimation of *ET*_o_ using the FAO56-PM method

The FAO56-PM equation for estimating *ET*_o,s_ is written as bellow (Allen *et al*.)^5^:

1 $$E{T}_{{\rm{O}},{\rm{S}}}=\frac{0.408{\rm{\Delta }}({R}_{{\rm{n}}}-G)+\gamma \frac{900}{{T}_{{\rm{ave}}}+273}{U}_{{\rm{2}}}({e}_{{\rm{s}}}-{e}_{{\rm{a}}})}{{\rm{\Delta }}+\gamma (1+0.34{U}_{{\rm{2}}})}$$

where *G* is soil heat flux (MJ m^−2^ month^−1^), *T*_ave_ is mean air temperature at 2 m (°C), ${T}_{ave}=({T}_{{\rm{\max }}}+{T}_{{\rm{\min }}})/2$, *U*_2_ and *U*_10_ are wind speed at 2 and 10 m (m s^−1^), respectively, *U*_2_ = 0.75 *U*_10_, ${e}_{{\rm{s}}}$ is saturation vapor pressure (kpa), *e*_a_ is actual vapor pressure (kpa), *e*_s_-*e*_a_ is saturation vapor pressure deficit (kpa), Δ is slope of vapor pressure curve (kpa °C^−1^), *γ* is psychrometric constant (kpa °C^−1^), and *R*_n_ is net radiation (MJ m^−2^ month^−1^). Monthly *G* is estimated by:

2 $${G}_{{\rm{K}}}=0.07({T}_{K+1}-{T}_{{\rm{K}}-{\rm{1}}})$$

where subscripts *K* + 1, *K* and *K* − 1 are order of month, respectively. Annual *ET*_o,s_ is cumulated from the values of 12 months.

*R*_n_ is calculated by:

3 $${R}_{{\rm{n}}}={R}_{{\rm{ns}}}-{R}_{{\rm{nl}}}$$

4 $${R}_{{\rm{ns}}}=(1-\alpha ){R}_{{\rm{s}}}$$

5 $${R}_{{\rm{s}}}=[{a}_{{\rm{s}}}+{b}_{{\rm{s}}}(\frac{n}{N})]{R}_{{\rm{a}}}$$

6 $${R}_{{\rm{nl}}}=\sigma (\frac{{T}_{\max \,,k}^{4}+{T}_{\min \,,k}^{4}}{2})(0.34-0.14\sqrt{{e}_{{\rm{a}}}})(1.35\frac{{R}_{{\rm{s}}}}{{R}_{{\rm{so}}}}-0.35)$$

where *R*_ns_ is net shortwave radiation (MJ m^−2^ month^−1^), *R*_nl_ is net longwave radiation (MJ m^−2^ month^−1^), *n* and *N* are actual and maximum possible sunshine duration, respectively, *R*_a_ is the extraterrestrial radiation (MJ m^−2^ month^−1^), *σ* is the Stefan-Boltzmann constant (4.903 × 10^−9^ MJ K^−4^ m^−2^ d^−1^), *α* is albedo (*α* = 0.23), *T*_max,k_ and *T*_min,k_ are maximum and minimum absolute temperatures during 24-h, respectively, and *R*_so_ is clear sky solar radiation (MJ m^−2^ month^−1^). The FAO56-PM recommended 0.25 for *a*_s_ and 0.50 for *b*_s_, respectively. For better accuracy, the calibrated values of *a*_s_ and *b*_s_ at 48 sites reported by Chen *et al*.^62^ were used here (marked with red triangle) for determination of *a*_s_ and *b*_s_ values at nearby sites (marked with blue circle in Fig. 1b) using the Thiessen polygon method.

### Estimation of *ET*_o_ using the other 10 selected methods

A preliminary performance comparison of 16 *ET*_o_ (*ET*_p_) methods were conducted (Fig. S1). From the elementary results, *ET*_p_ equations performed generally worse than *ET*_o_ equations. Therefore, 10 *ET*_o_ equations which performed generally well in different regions of the world, i.e., Irmak *et al*.^65^, Makkink^66^, Priestley-Taylor^23^, Hargreaves-Samani^16^, Droogers-Allen^67^, Berti *et al*.^68^, Doorenbos-Pruitt^4^, Wright^69^ and Valiantzas^14^, are selected to compare to the FAO56-PM equation. Of which, Valiantzas^14^ proposed two equations to simplify the FAO56-PM equation. The two Valiantzas^14^ equations and the Berti *et al*.^68^ equation were relatively new, but their performances have not been validated in China. Three Hargreaves-Samani-based equations (HS, MHS_1 and MHS_2) are adopted here because the FAO-56 manual recommended HS as the use of a less demanding method with only data on *T*_ave_ and extraterrestrial radiation (*R*_a_)^70^. The types, simplified method name, and main equations for estimating *ET*_o_ of the selected 10 methods (*ET*_o,i_, *i* = 1, 2, …, 10) are given in Table 1. For the Droogers and Allen^67^ method (simplified as MHS_1), ${S}_{o}=15.392{d}_{{\rm{r}}}({w}_{{\rm{s}}}\,\sin (\phi )\,\sin (\delta )+\,\cos (\phi )\,\cos (\delta )\,\sin ({w}_{{\rm{s}}}))$. For the Berti *et al*.^68^ method (simplified as MHS_2), *P* is precipitation. For the Valiantzas^14^ method (simplified as Val_1), *T*_dew_ = [116.91 + 237.3 ln(e_a_)]/[16.78-*ln*(e_a_)]. For the Wright^69^ method (simplified as KPM), *a*_w_ = 0.3 + 0.58 exp $[-{(\frac{J-170}{45})}^{2}]$, *b*_w_ = 0.32 + 0.54 exp $[-{(\frac{J-228}{67})}^{2}]$, where *J* is Julian day in the year between 1 (1 January) and 365 or 366 (31 December).

**Table 1:** Equations of *ET*_o,i_ estimated with the selected 10 methods.

*i*	Proposed by	Simplified as	Equation	Type
1	Irmak *et al*. (2003)	IRA	*ET*_o,1_ = 0.489 + 0.289 *R*_n_ + 0.023 *T*_ave_	Empirical-based
2	Makkink^66^	Mak	*ET*_o,2_ = 0.61(1/λ) [Δ/(Δ + γ)]*R*_s_ − 0.12	Radiation-based
3	Priestley-Taylor^23^	PT	*ET*_o,3_ = 1.26[Δ/(Δ + γ)] (*R*_n_ − *G*)/λ	
4	Hargreaves-Samani^16^	HS	*ET*_o,4_ = [0.0023 *R*_a_(*T*_ave_ + 17.8) (*T*_max_ − *T*_min_)^0.5^]/λ	Temperature-based
5	Droogers and Allen^67^	MHS_1	*ET*_o,5_ = 0.0013 *S*_o_(*T*_ave_ + 17) [(*T*_max_ − *T*_min_) − 0.0123 *P*]^0.76^	
6	Berti *et al*.^68^	MHS_2	*ET*_o,6_ = [0.00193 *R*_a_(*T*_ave_ + 17.8) (*T*_max_ − *T*_min_)^0.517^]/λ	
7	Doorenbos-Pruitt^4^	FAO24	*ET*_o,7_ = [$\tfrac{{\rm{\Delta }}}{{\rm{\Delta }}+\gamma }$(*R*_n_ − *G*) + 2.7$\tfrac{\gamma }{\gamma +{\rm{\Delta }}}$(1 + 0.864 *U*_2_) (*e*_s_ − *e*_a_)]/λ	Combination
8	Wright^69^	KPM	*ET*_o,8_ = [$\tfrac{{\rm{\Delta }}}{{\rm{\Delta }}+\gamma }$(*R*_n_ − *G*) + 6.43$\tfrac{\gamma }{\gamma +{\rm{\Delta }}}$(*a*_w_ + *b*_w_ *U*_2_) (*e*_s_ − *e*_a_)]/λ	
9	Valiantzas^14^	Val_1	*ET*_o,9_ = 0.00668 *R*_a_[(*T*_ave_ + 9.5) (*T*_max_ − *T*_dew_)]^0.5^ − 0.0696(*T*_max_ − *T*_dew_) − 0.024(*T*_ave_ + 20) (1 − *RH*) − 0.00455 *R*_a_(*T*_max_ − *T*_dew_)^0.5^ + 0.0984(*T*_ave_ + 17) [1.03 + 0.00055(*T*_max_ − *T*_min_)^2^ − *RH*]	Simplified FAO56-PM
10	Valiantzas^14^	Val_2	*ET*_o,10_ = 0.03825 *R*_s_(*T*_ave_ + 9.5)^0.5^ − 2.4(*R*_s_/*R*_a_)^2^ + 0.048(*T*_ave_ + 20) (1 − *RH*) (0.5 + 0.536 *U*_2_) + 0.00012 *altitude*	

### Performance evaluation of the 10 selected methods

Relative error (*RE*), standard deviation (*θ*) and Nash-Sutcliffe efficacy coefficient (*NSE*)^71^ are used to assess the performances of monthly *ET*_o,i_:

7 $$RE=\frac{E{T}_{{\rm{o}},{\rm{i}}}-E{T}_{o,s}}{E{T}_{o,s}}$$

8 $$\theta =\sqrt{\frac{\sum {({x}_{{\rm{i}}}-\overline{x})}^{2}}{N-1}}$$

9 $$NSE=1-\frac{{\sum }_{i=1}^{n}{(E{T}_{o,s}-E{T}_{o,i})}^{2}}{{\sum }_{i=1}^{n}{(E{T}_{o,s}-\overline{E{T}_{o,s}})}^{2}}$$

where N = 1, 2, …, 636th month. If *RE* is close to 0, *ET*_o,i_ is close to *ET*_o,s_. The *NSE* values ranged from −∞ to 1. When *NSE* is close to 1, the quality of the method for estimating *ET*_o,i_ is good with high reliability. When *NSE* is close to 0, *ET*_o,i_ has an close mean value with *ET*_o,s_ with an overall reliable estimation, but the errors of the estimation processes are large; when *NSE* is much less than 0, the estimation is not reliable.

### Trend test

The modified nonparametric Mann-Kendall (MMK) test^72^, which takes into account the effects of auto-correlation in annual time series *ET*_o,L_ (*L* = 1, 2, …, *n*_1_, where *n*_1_ = 53 is total year number) based on the standardized Mann-Kendall (MK) method^73, 74^, is used to test the trend of *ET*_o,L_ if it is auto-correlated^8^. The MK test statistic (*Z*) follows the standard normal distribution with a mean of 0 and variance of 1 under the null hypothesis of no trend in *ET*_o,L_. The null hypothesis is rejected if |*Z*| ≥ *Z*_1-β/2_ at a confidence level of *β*, where *Z*_1-*β*/2_ is the (1-*β*/2)–quantile. If *Z* is positive (or negative), *ET*_o,L_ has an upward (or downward) trend. As *β* = 0.05, if |*Z*| > 1.96, the trend is significant. The MMK statistic *Z* ^*^ is computed by introducing a correction factor ${n}_{1}^{s}$ to *Z* to estimate^72^:

10 $${Z}^{\ast }=Z/\sqrt{{n}_{1}^{s}},\,{\rm{where}}\,{n}_{1}^{s}=\{\begin{array}{ll}1+\tfrac{2}{{n}_{1}}\sum _{jj=1}^{{n}_{1}-1}({n}_{1}-1){r}_{jj} & for\,jj > 1\\ 1+2\tfrac{{r}_{1}^{{n}_{1}+1}-{n}_{1}{r}_{1}^{2}+({n}_{1}-1){r}_{1}}{{n}_{1}{({r}_{1}-1)}^{2}} & for\,jj=1\end{array}$$

where *r*_jj_ is sample autocorrelation coefficient of *ET*_o,L_ at a lag *jj*. For denoting significance of a trend, when *jj* = 0, *Z* ^*^ equals to *Z*; while as *jj* > 0, the MMK statistic *Z* ^*^ is utilized.

### Variation coefficient

The variability of series *ET*_o,L_ is quantified with a coefficient of variation (*C*_v_), calculated with the following equation (Nielsen and Bouma)^75^:

11 $${C}_{v}=\frac{\theta }{\overline{E{T}_{{\rm{o}},L}}}\,{\rm{or}}\,{C}_{v}=\frac{\theta }{\overline{E{T}_{{\rm{o}},S}}}$$

where *θ* and $\overline{E{T}_{{\rm{o}},L}}$ are standard deviation and multi-year mean *ET*_o,L_ series, respectively. Variability levels are classified by *C*_v_ ≤ 0.1, 0.1 < *C*_v_ < 1.0 and *C*_v_ ≥ 1.0 as weak, moderate or strong one, respectively.

Spatial distributions of the climatic variables, *ET*_o,s_, *ET*_o,i_ and the other studied parameters are mapped by the Kriging interpolation method in ArcGIS 10.2 software.

## Results

### Spatial distribution of climatic variables

**Figure 2:**
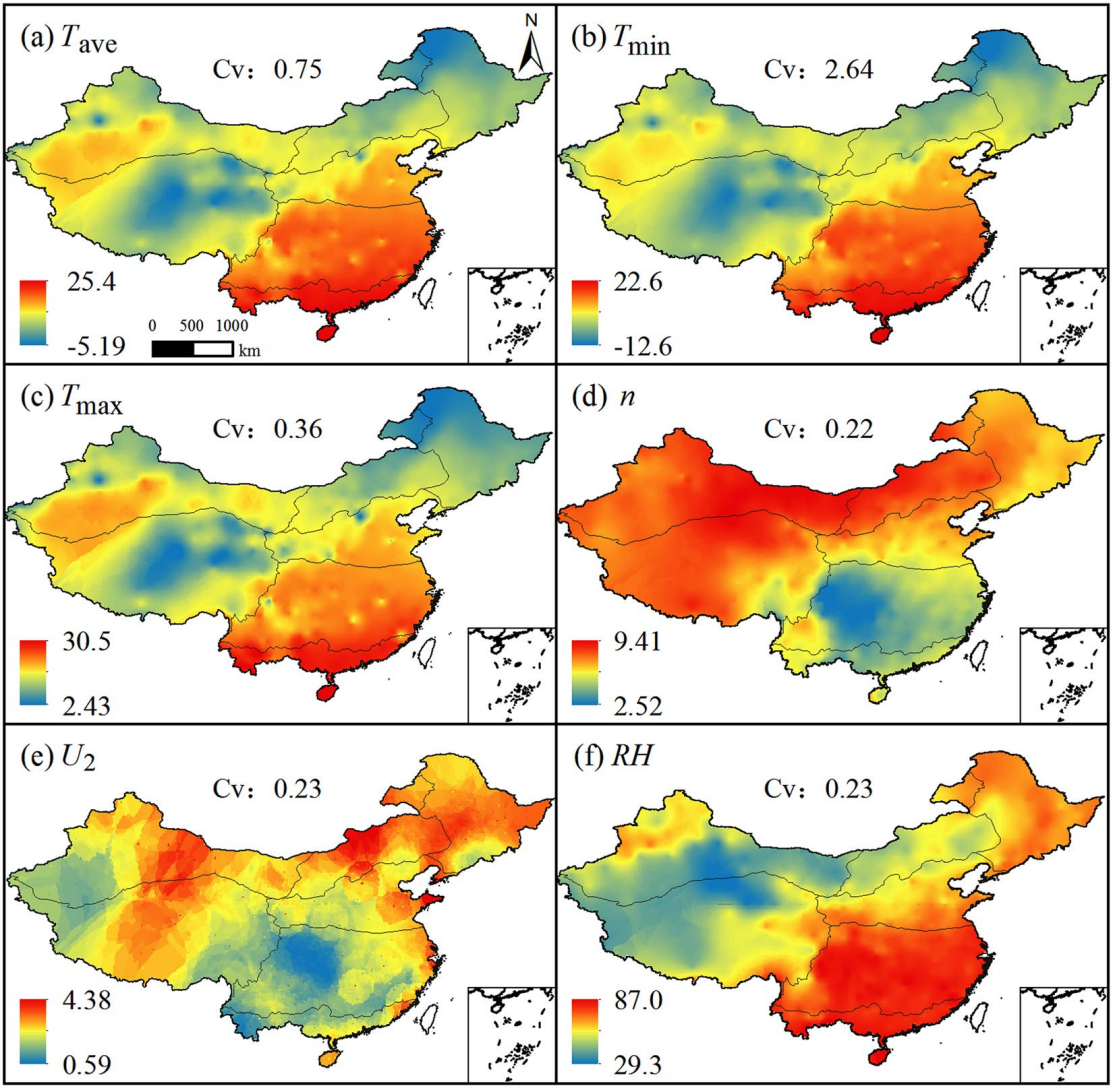
Spatial distributions of multi-year mean meteorological elements in China. (ArcGIS 10.2, http://map.baidu.com, Lingling Peng).

The spatial distribution of *ET*_o_ are closely related to that of the related meteorological elements. Figure 2 illustrates the distribution of multi-year mean *T*_ave_, *T*_min_, *T*_max_, *n*, *U*_2_ and *RH*. The distribution of *T*_min_, *T*_max_, *T*_ave_ were generally similar, with high values in sub-regions V and VII but lower values in sub-regions II, III and VI. Values of *n* were higher in sub-regions I, II, III and VI. *U*_2_ values were large in north China especially in sub-regions II and III, and small in sub-region V, generally. Values of *RH* were higher in the southeast China for sub-regions V and VII. *T*_ave_, *T*_max_, *n*, *U*_2_ and *RH* showed moderate variability, while *T*_min_ showed strong variability.

### Spatial distribution of *ET*_o,s_ and its trend

The equations and types of the FAO56-PM (for estimating *ET*_o,s_) and the other 10 methods (for estimating *ET*_o,i_) are shown in Table 1. Detail description of the equations is in the section “Data and methodology”.

**Figure 3:**
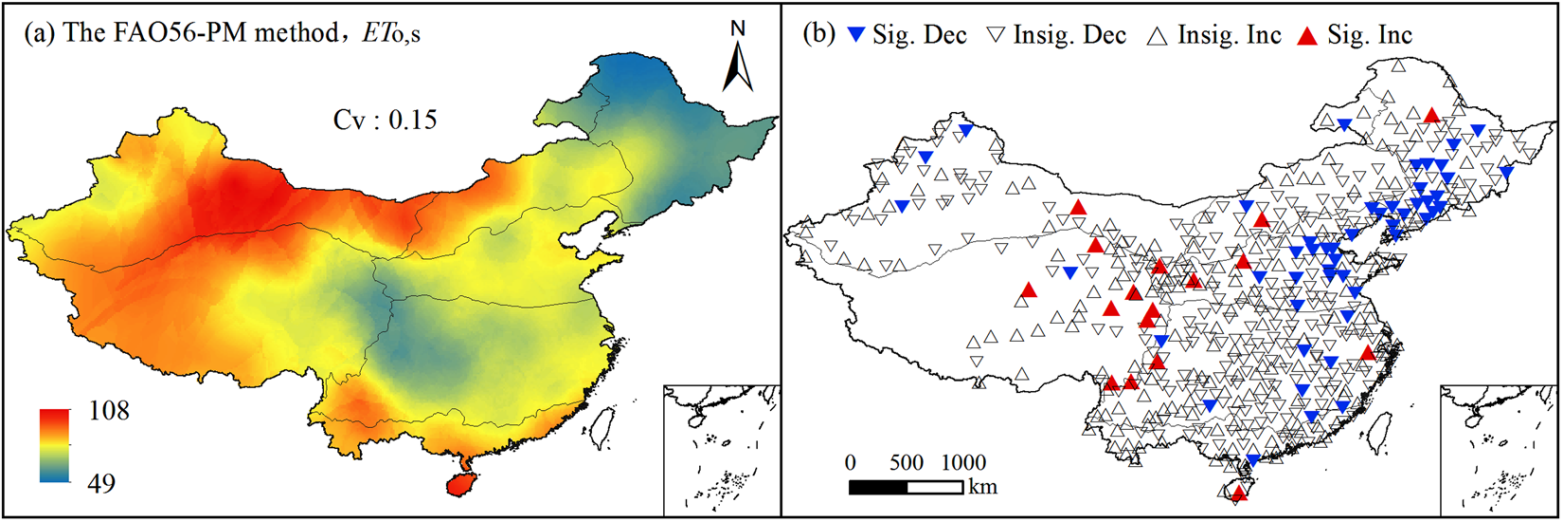
Spatial distribution of multi-year mean monthly *ET*_o,s_ and the annual *ET*_o,s_ trends over 1961–2013 at the 552 sites in mainland China. (ArcGIS 10.2, http://map.baidu.com, Lingling Peng).

Figure 3 shows the spatial distribution of multi-year mean monthly *ET*_o,s_ and trend test results of annual *ET*_o,s_ series for each site. The site number for different trends is presented in Table 2. In Fig. 3a, the *ET*_o,s_ values were higher in the sub-regions I, II, VI and VII than the other 3 sub-regions, ranging from 49 to 108 mm. *ET*_o,s_ had a moderate spatial variability with a coefficient of variation (*C*_v_) being 0.15. In general, *ET*_o,s_ in western China (high elevations) were larger than in eastern and middle China (low elevations). In Fig. 3b and Table 2, more sites (339) had decrease trends in *ET*_o,s_ than increase trends (213 sites), and the trends at more sites were insignificant. The sites which had decrease and increase trends occupied 61.4% and 38.6% of the total, respectively. This indicated an overall decrease of *ET*_o,s_ in China. The common occurrence of insignificance trends was induced by the removing of autocorrelation structures when using the modified nonparametric Mann-Kendall test (MKK) method. It’s reasonable for the trend analysis. The sites with significant decrease (Sig. Dec) trends in *ET*_o,s_ were mainly located in eastern China, while the sites with significant increase (Sig. Inc) trends in *ET*_o,s_ were mainly located in middle China (i.e., sub-region VI).

**Table 2:** Number of the sites with different trends (tested by the MMK method) for the annual *ET*_o_ series over the period 1961–2013 in different sub-regions of China.

Sub-region	Sig. Dec	Insig. Dec	Sig. Inc	Insig. Inc
I	3	36	1	21
II	2	23	1	18
III	16	36	1	19
IV	18	54	3	29
V	8	99	2	56
VI	1	15	8	25
VII	1	27	1	28
EMC	49	290	17	196

Sig. Dec-significant decrease, Insig. Dec-insignificant decrease, Sig. Inc-significant increase, Insig. Inc-insignificant increase.

### The Spatiotemporal variation of *ET*_o,i_

**Figure 4:**
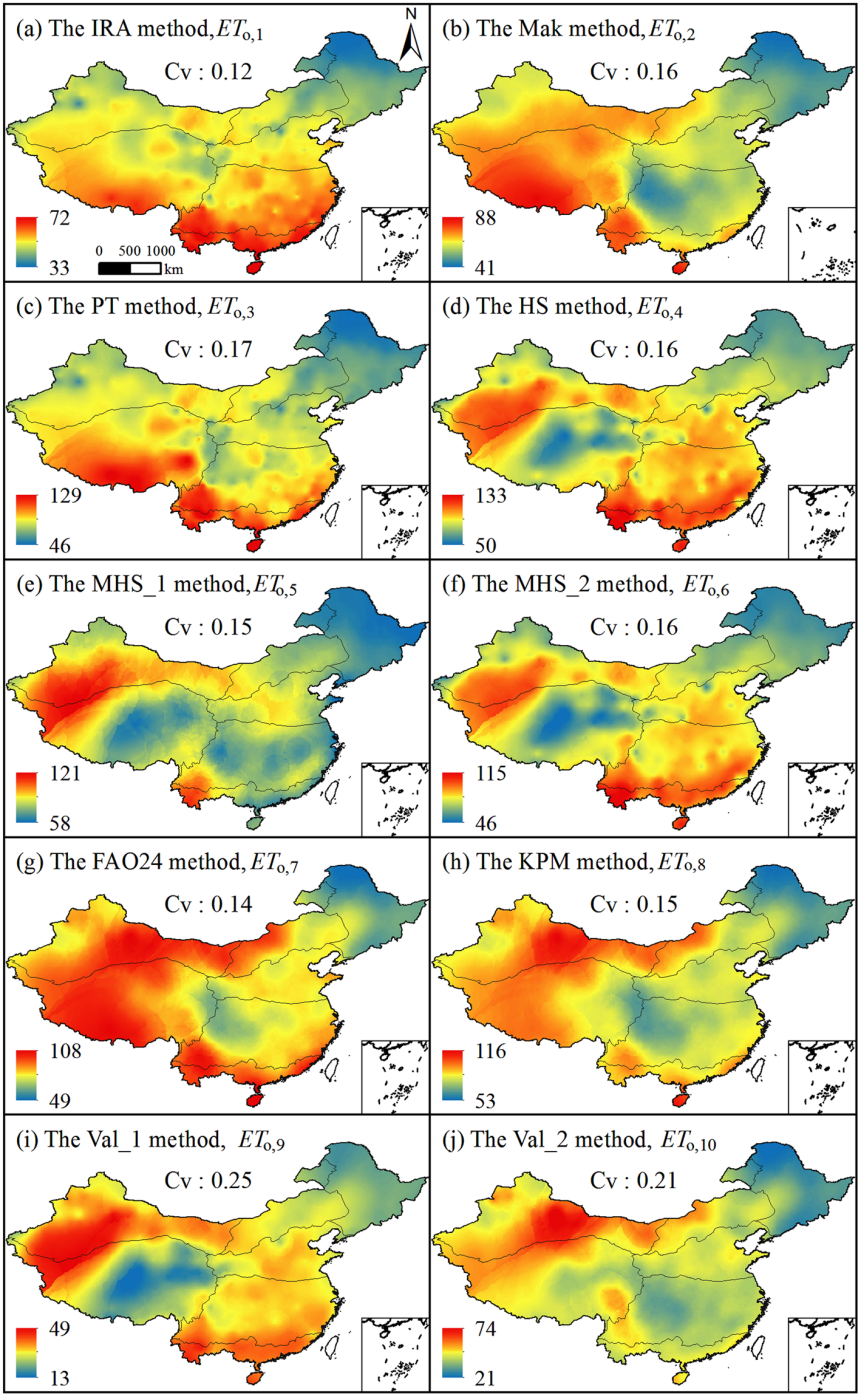
Spatial distribution of multi-year mean monthly *ET*_o,i_ in China. (ArcGIS 10.2, http://map.baidu.com, Lingling Peng).

The spatial distribution of multi-year mean monthly *ET*_o,i_ values during 1961–2013 showed remarkable differences between different sub-regions, their variation ranges and the spatial distributions of *ET*_o,i_ also had different similarity with *ET*_o,s_ (Fig. 4). All of the 10 methods had different ranges of *ET*_o,i_, obtained lower *ET*_o_ values in the northeastern China (sub-region III), and differed much in spatial distribution when compared with *ET*_o,s_. The empirical method for estimating *ET*_o,1_ only resembled *ET*_o,s_ distribution in sub-region III very well, and its ranges were much smaller than *ET*_o,s_. *ET*_o,2_ and *ET*_o,3_ (radiation-based) distributed partly similar with *ET*_o,s_. Among the temperature-based methods for estimating *ET*_o_ (i.e., *ET*_o,4_, *ET*_o,5_ and *ET*_o,6_), *ET*_o,6_ resembled the spatial distribution and the value range of *ET*_o,s_ more. The spatial distribution of *ET*_o,7_ and *ET*_o,8_ (combination type) were highly similar with that of *ET*_o,s_. *ET*_o,9_ and *ET*_o,10_ (simplified FAO56-PM) had high similarity in spatial distribution with *ET*_o,s_, but with much smaller ranges than *ET*_o,s_. *ET*_o,i_ generally had moderate variability (*C*_v_ < 1.0), of which, *C*_v_ values of *ET*_o,9_ and *ET*_o,10_ were the first and the next largest, *C*_v_ values of *ET*_o,1_ to *ET*_o,8_ were small.

**Figure 5:**
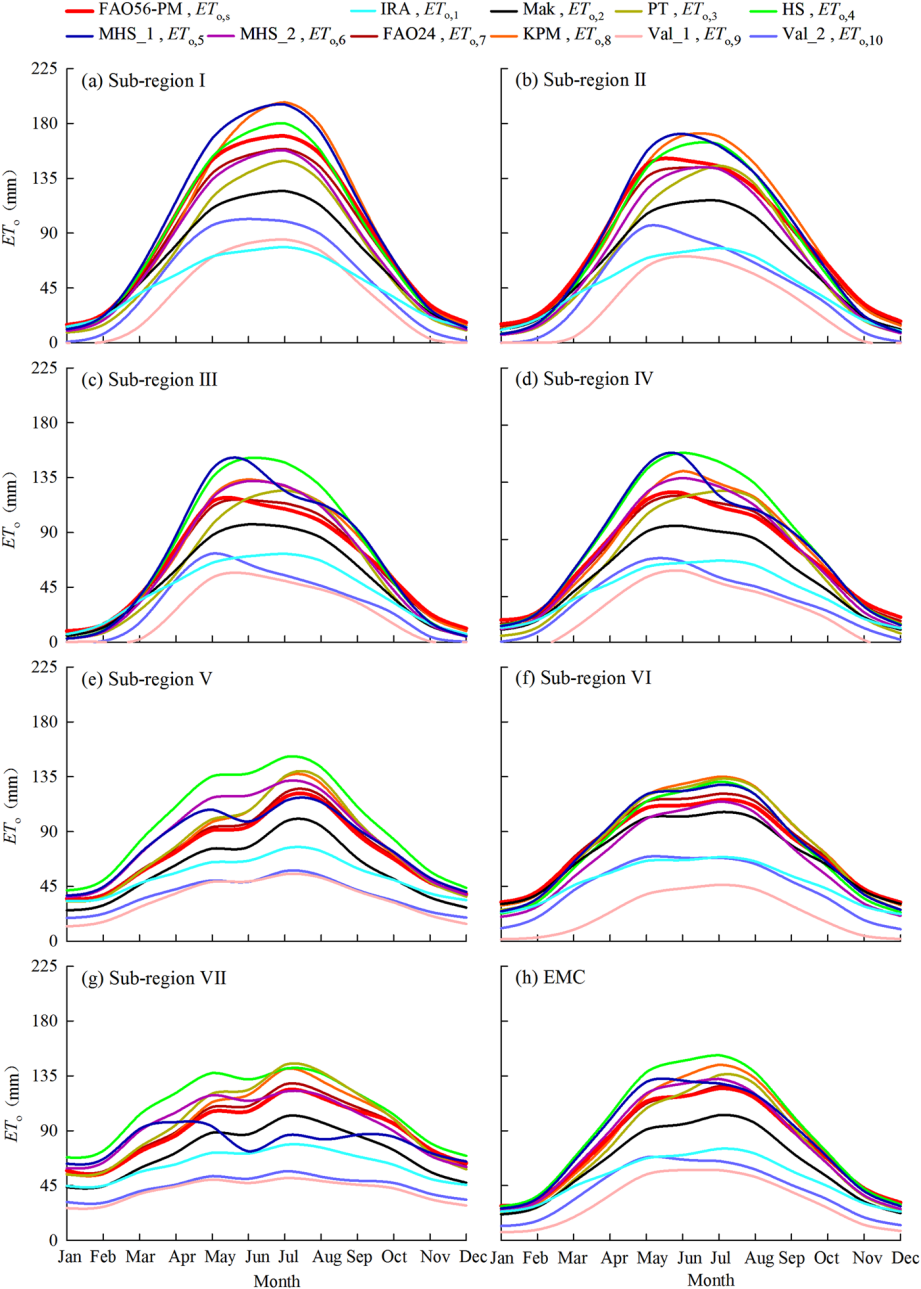
Temporal variations of multi-year mean monthly *ET*_o,s_ and *ET*_o,i_ in different sub-regions and EMC.

**Figure 6:**
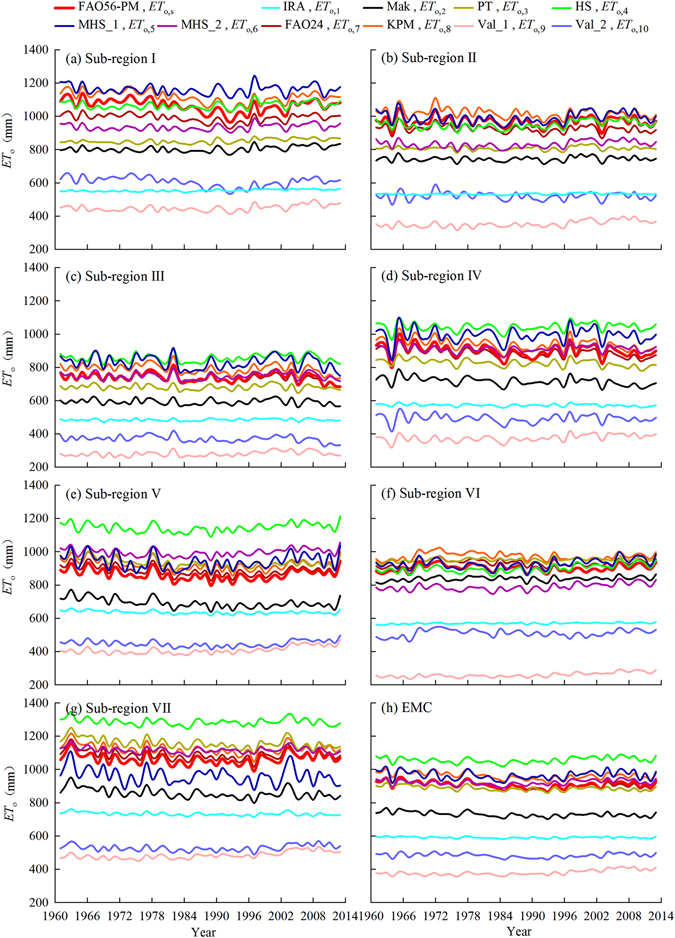
The inter-annual variations of *ET*_o,i_ and *ET*_o,s_ in different sub-regions and EMC over 1961–2013.

The temporal variations of multi-year mean monthly and annual *ET*_o,i_ in different sub-regions showed various similarity with that of *ET*_o,s_ (Figs 5 and 6). In Fig. 5, the variation patterns of monthly *ET*_o,i_ and *ET*_o,s_ were general with single peak (valley) around July (January or December), which were also the months that the largest (smallest) differences between *ET*_o,i_ and *ET*_o,s_ occurred. The differences between *ET*_o,i_ and *ET*_o,s_ curves was the largest for the sub-region I (northwestern China), and was the smallest for the sub-region VI (the Qinghai-Tibetan Plateau). The *ET*_o,s_ curves were generally in the upper of the 11 curves for different sub-regions. Of the ten curves, *ET*_o,1_, *ET*_o,2_, *ET*_o,9_ and *ET*_o,10_ deviated *ET*_o,s_ much and were not suitable for best alternative of *ET*_o,s_. *ET*_o,7_ estimated by the FAO24 method had the smallest differences in all of the 7 sub-regions and EMC, followed by *ET*_o,8_ estimated by the Wright^69^ method. The other *ET*_o,i_ (*i* = 3, 4, 5, and 6) curves differed but had neither the largest nor the smallest deviations with *ET*_o,s_ curves. *ET*_o,4_ curves for sub-region I and *ET*_o,7_ for sub-regions II to VII and EMC were closest to *ET*_o,s_ curve. Except *ET*_o,7_ which was a combination type estimated with a high data-requirement, *ET*_o,6_ for sub-regions I, II, IV, VI and EMC were also very close to *ET*_o,s_ curve. *ET*_o,6_ was estimated by the modified Hargreaves-Samani (Berti *et al*.^68^), which belonged to the temperature-based type, needed only temperature data, and was simple in computation. In general, both *ET*_o,i_ and *ET*_o,s_ curves were regional-, seasonal- and method-specific.

In Fig. 6, the annual variations of *ET*_o,i_ or *ET*_o,s_ generally had similar temporal variation patterns over 1961–2013 but their values differed a lot. For sub-region I, *ET*_o,i_ curves ranked with a method order of MHS_1 > KPM > FAO56-PM > HS > FAO24 > MHS_2 > PT > Mak > Val_2 > IRA > Val_1, i.e., *ET*_o,5_ > *ET*_o,8_ > *ET*_o,s_ > *ET*_o,4_ > *ET*_o,7_ > *ET*_o,6_ > *ET*_o,3_ > *ET*_o,2_ > *ET*_o,10_ > *ET*_o,1_ > *ET*_o,9_, while the orders changed for the other sub-regions. Differences between annual *ET*_o,i_ and *ET*_o,s_ curves were generally large in sub-regions I and VII, but small in sub-regions III and VI. For sub-regions III, IV and EMC, annual *ET*_o,6_ values were much close to *ET*_o,s_. For the other sub-regions, annual *ET*_o,7_ was also similar to *ET*_o,s_. Annual *ET*_o,1_ *ET*_o,2_, *ET*_o,9_ and *ET*_o,10_ values were much smaller at most sub-regions and EMC, which was similar to the results of monthly *ET*_o,1_ *ET*_o,2_, *ET*_o,9_ and *ET*_o,10_. Also, both annual *ET*_o,i_ and *ET*_o,s_ curves were regional-, seasonal-, and method-specific.

### Performance comparison of the selected 10 methods for estimating *ET*_o,i_

#### Relative error

**Figure 7:**
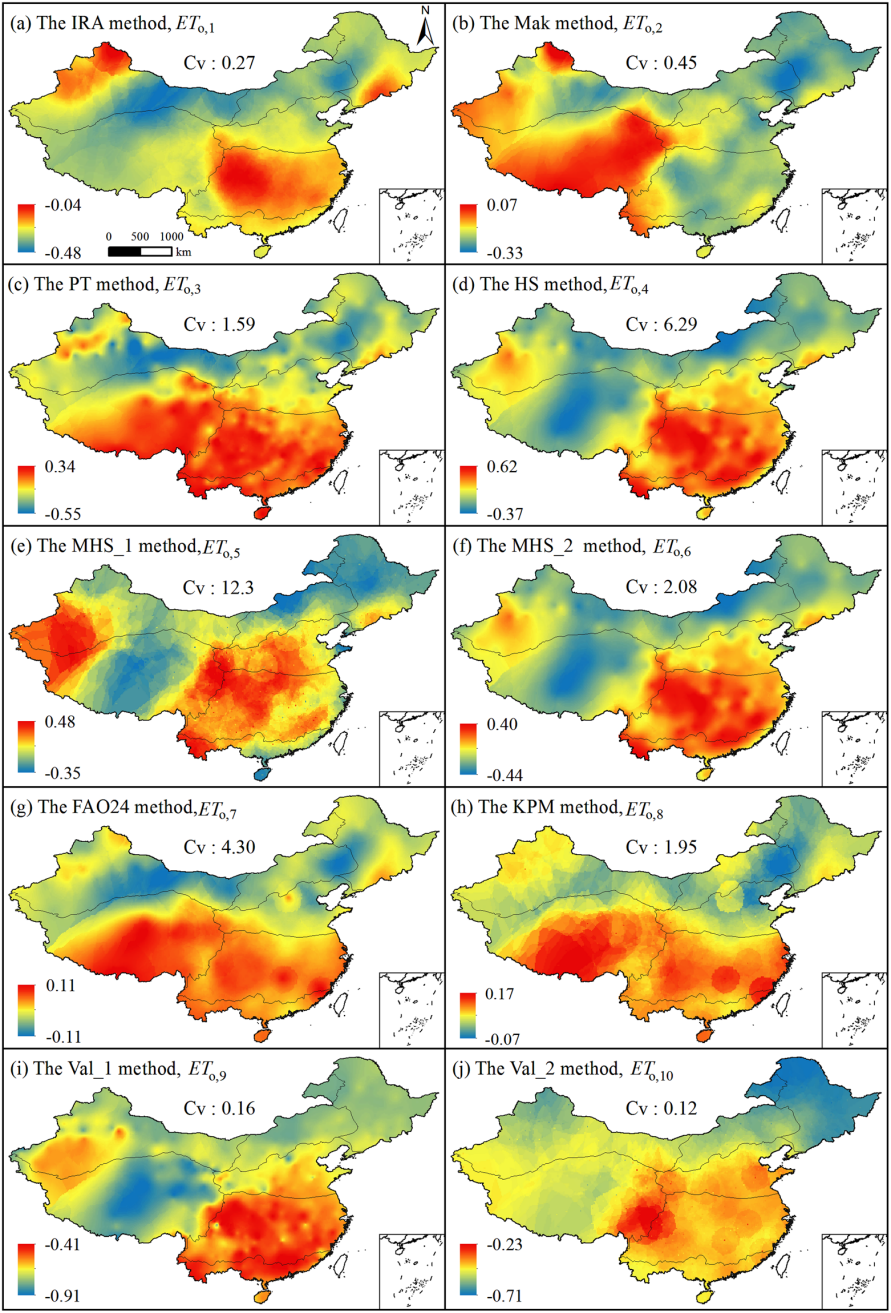
Spatial distribution of *RE* values for multi-year mean monthly *ET*_o,i_ in EMC. (ArcGIS 10.2, http://map.baidu.com, Lingling Peng).

Because the estimated *ET*_o,i_ values were regional-specific, the *RE* values for *ET*_o,i_ also showed differences in spatial distributions (Fig. 7). Ranges of *RE* for *ET*_o,i_ varied. The range of absolute *RE* values for *ET*_o,9_ was the largest, followed by *ET*_o,10_. *RE* for most of *ET*_o,i_ covered both negative and positive values, but *RE* range of *ET*_o,1_, *ET*_o,9_ and *ET*_o,10_ covered only negative values. *ET*_o,7_ had the smallest *RE* range, which reflected that the FAO24 method was more accurate for estimating monthly *ET*_o_ in EMC. The radiation-based Mak method had smaller *RE* ranges when compared to the empirical, temperature-based methods and another radiation-based method PT, but it generally underestimated *ET*_o_ in most of the months and sites and only had local adaptability in sub-region VI, therefore this method shouldn’t be the best alternative for *ET*_o,s_ in different sub-regions and EMC. Considering the simpler temperature-based *ET*_o_ type, the MHS_2 method had lower *RE* than the other temperature-based methods. In general, the spatial distribution of *RE* for different *ET*_o,i_ differed at different locations. It revealed the differences in adaptability extents of the applied methods.

**Figure 8:**
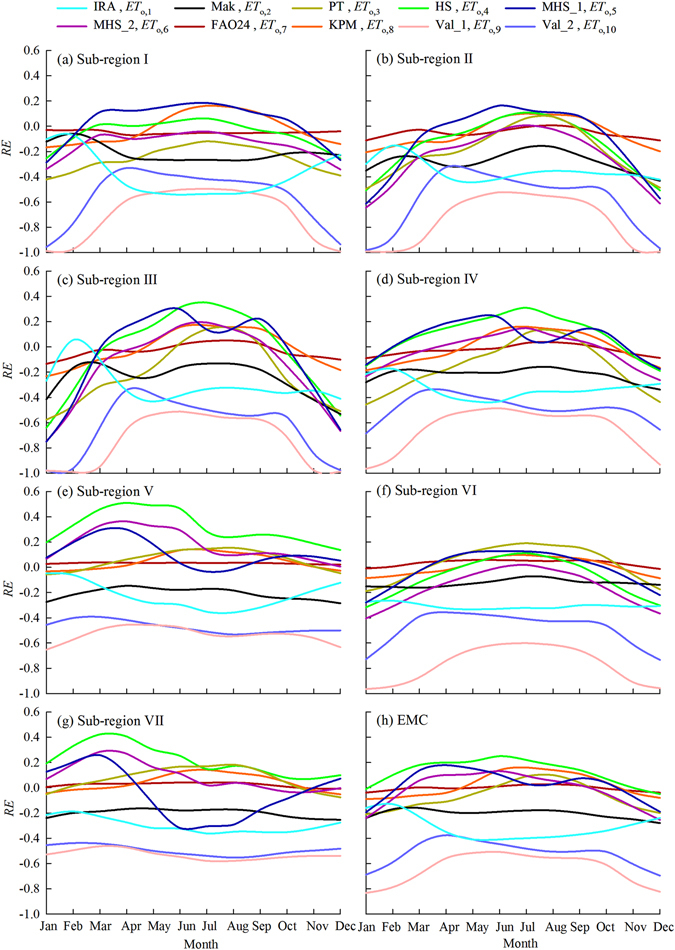
The temporal variations of *RE* values for multi-year mean monthly *ET*_o,i_ in different sub-regions and EMC.

Generally consistent with the spatial distribution, the temporal variations of *RE* for monthly *ET*_o,i_ were also method and sub-region-specific (Fig. 8). The largest (smallest) *RE* curves generally occurred for *ET*_o,9_ (*ET*_o,7_) in all of the 7 sub-regions and EMC. The largest negative *RE* curves were *ET*_o,1_, *ET*_o,9_ and *ET*_o,10_ in all of the 7 sub-regions and EMC, indicating worse performance of the methods IRA, Val_1 And Val_2. Although generally varied with the month, *RE* values for *ET*_o,4_, *ET*_o,6_, *ET*_o,7_, and *ET*_o,8_ ranged between −0.2 to 0.2 in most time of the year for 3, 4, 7 and 7 sub-regions, respectively. The *RE* values were generally small for EMC when compared to any one of the sub-regions or the methods. In general, in the temperature-based methods, MHS_2 performed the best in most time of the year for most of the sub-regions.

The relative error (*RE*) values of the monthly and annual *ET*_o,i_ using the selected 10 methods for EMC are presented in Table 3. Values of *ET*_o,1_, *ET*_o,2_, *ET*_o,9_ and *ET*_o,10_ underestimated *ET*_o,s_ in all the 12 months and the whole year, of which, both monthly and annual *ET*_o,9_ had the largest deviations, followed by *ET*_o,10_. *ET*_o,3_ underestimated *ET*_o,s_ in 8 months (except June, July, August, September) and the whole year. *ET*_o,8_ underestimated *ET*_o,s_ in 6 months in January, February, March, April, November, December but slightly overestimated annual *ET*_o,s_. Moreover, the *RE* values were mostly month-free (i.e., overall larger or smaller than *ET*_o,s_ in most months of the year) when comparing different estimation methods. However, *ET*_o,4_, *ET*_o,5_, *ET*_o,6_, *ET*_o,7_ and *ET*_o,8_ overestimated *ET*_o,s_ in 10, 8, 7, 7 and 6 months of the year, respectively, which resulted to overestimated annual *ET*_o_. The *RE* values for annual *ET*_o,i_ ranked in an order of *ET*_o,9_ > *ET*_o,10_ > *ET*_o,1_ > *ET*_o,2_ > *ET*_o,4_ > *ET*_o,3_ > *ET*_o,5_ > *ET*_o,6_ > *ET*_o,8_ > *ET*_o,7_, corresponding to the method order of Val_1 > Val_2 > IRA > Mak > HS > PT > MHS_1 > MHS_2 > KPM > FAO24. Each method overestimated or underestimated *ET*_o_ in different months or the whole year when compared to FAO56-PM, but in the temperature type, the MHS_2 method was found to be the closest to FAO56-PM considering. Although the MHS_2 method underestimated the *ET*_0,s_ by 24% in January, and 15% and 25% in November and December, but had a very low *RE* (2%) for the year when compared to the FAO56-PM method.

**Table 3:** Relative error (*RE*) values of the 10 selected methods for estimating *ET*_o,i_ at the monthly and annual timescales for China.

*ET*_o,i_	*ET*_o,1_	*ET*_o,2_	*ET*_o,3_	*ET*_o,4_	*ET*_o,5_	*ET*_o,6_	*ET*_o,7_	*ET*_o,8_	*ET*_o,9_	*ET*_o,10_
Month/Year										
Jan	−0.16	−0.24	−0.21	−0.01	−0.19	−0.24	−0.04	−0.09	−0.83	−0.69
Feb	−0.13	−0.17	−0.18	0.10	−0.03	−0.09	−0.02	−0.08	−0.79	−0.59
Mar	−0.23	−0.16	−0.13	0.18	0.14	0.06	0.01	−0.06	−0.68	−0.44
Apr	−0.35	−0.19	−0.11	0.20	0.18	0.10	0.01	−0.04	−0.56	−0.38
May	−0.41	−0.20	−0.05	0.21	0.15	0.11	0.01	0.04	−0.52	−0.41
Jun	−0.41	−0.19	0.02	0.25	0.10	0.13	0.01	0.14	−0.51	−0.45
Jul	−0.40	−0.18	0.09	0.22	0.03	0.09	0.02	0.16	−0.54	−0.48
Aug	−0.39	−0.18	0.10	0.18	0.03	0.06	0.03	0.14	−0.55	−0.51
Sep	−0.37	−0.20	0.04	0.14	0.07	0.02	0.02	0.11	−0.56	−0.50
Oct	−0.34	−0.23	−0.06	0.07	0.04	−0.05	−0.01	0.04	−0.61	−0.51
Nov	−0.29	−0.25	−0.15	0.00	−0.07	−0.15	−0.02	−0.04	−0.75	−0.61
Dec	−0.24	−0.28	−0.20	−0.05	−0.19	−0.25	−0.04	−0.08	−0.82	−0.70
Year	−0.35	−0.20	−0.02	0.16	0.06	0.02	0.01	0.06	−0.58	−0.47

#### Standard deviation

The spatial distribution of multiyear mean monthly standard deviation (*θ*) for *ET*_o,i_ are illustrated in Fig. S2. All of the ten *ET*_o_ estimation methods showed larger *θ* values in the northern China (sub-regions I, II and III), although with different ranges of *θ*. There was a method order of ranges for *θ*, i.e., IRA < Val_1 < Mak < PT < MHS_2 < HS < FAO24 < MHS_1 < Val_2 < KPM. In general, a larger ranges of *ET*_o,i_ corresponded to a larger ranges of *θ*, the IRA method had a smallest range of *θ* because it had a smaller range of *ET*_o._ In fact, this method largely underestimated *ET*_o,s_. The KPM method had a largest range of *θ*, which indicated the variation scope of *ET*_o,8_ values was large. The index *θ* didn’t reflect the deviations of each *ET*_o,i_ to *ET*_o,s_, it only reflected the deviations of monthly *ET*_o,i_ to average *ET*_o,i_.

The temporal variations of standard deviation (*θ*) averaged for the 12 months for *ET*_o,i_ are illustrated in Fig. S3. The standard deviations of the monthly and annual *ET*_o_ in EMC are presented in Table 4. *θ* in sub-region I, VI and EMC were generally smaller than the other sub-regions for each month. The MHS_1 had the largest *θ* for all of the sub-regions and EMC. For EMC, the *θ* curves ranked in the method order of IRA < Val_1 < Val_2 < HS < Mak < PT < MHS_2 < FAO24 < KPM < MHS_1.

**Table 4:** Standard deviation values of *ET*_o,i_ (*i* = 1 to 10) and *ET*_o,s_ at the monthly timescale.

*ET*_o_	*ET*_o,1_	*ET*_o,2_	*ET*_o,3_	*ET*_o,4_	*ET*_o,5_	*ET*_o,6_	*ET*_o,7_	*ET*_o,8_	*ET*_o,9_	*ET*_o,10_	*ET*_o,s_
Month/Year											
Jan	0.84	2.15	0.93	2.46	3.23	2.17	1.69	1.49	0.79	1.07	2.19
Feb	1.23	2.86	1.28	4.23	5.12	3.72	2.10	1.89	1.63	2.03	2.69
Mar	1.32	3.55	2.59	4.68	6.13	4.13	3.28	3.05	2.54	2.84	3.94
Apr	1.17	3.34	2.60	4.46	6.27	3.95	3.28	3.17	2.95	2.84	4.14
May	1.13	3.26	2.99	3.91	6.99	3.49	4.06	4.48	2.80	3.57	4.94
Jun	1.24	3.57	3.74	3.48	6.63	3.11	4.23	5.18	2.49	2.81	4.75
Jul	1.53	4.16	4.75	3.16	6.40	2.82	4.52	5.50	2.40	2.50	4.65
Aug	1.50	4.12	4.50	3.11	5.83	2.77	4.31	5.36	2.33	2.45	4.48
Sep	0.98	2.78	2.46	2.85	5.18	2.54	2.70	3.37	2.11	1.74	3.04
Oct	0.89	2.96	1.72	2.90	5.02	2.59	2.20	2.42	2.17	1.45	2.65
Nov	0.98	2.57	1.12	2.82	4.06	2.50	1.80	1.75	1.50	1.44	2.42
Dec	0.91	2.19	0.92	2.36	3.17	2.09	1.57	1.41	0.84	1.05	2.09
Year	4.77	14.8	14.0	17.3	25.2	15.3	17.9	21.1	15.9	12.9	20.3

#### Nash-Sutcliffe efficiency coefficient

The spatial (temporal) distribution of multiyear mean monthly Nash-Sutcliffe efficiency coefficients (*NSE*s) for *ET*_o,i_ are illustrated in Figs S4 and S5. In Fig. S4, the ranges of *NSE* ranked in a method order of FAO24 < Mak < KPM < MHS_2 < PT < HS < MHS_1 < IRA < Val_2 < Val_1. The FAO24 and KPM, as analyzed above, were both combination based *ET*_o_ methods, although both had smaller *NSE* ranges, their equations had higher demand of climatic variables. The Mak method had a smaller range of *NSE* than MHS_2 (between −9.32 and 0.35), it performed better than MHS_2 in sub-regions IV and VI, but it needed addition shortwave radiation (or sunshine hour) when estimating *ET*_o_. The *NSE* of MHS_2 method ranged between −16 and 0.20, it performed well for most sub-regions except VI and VII. From climatic variable demand aspect, the MHS_2 best met a simple equation standard than the other equations, with general good performance. In Fig. S5, the Val_2, Val_1, IRA, HS and Mak were excluded from the ten *ET*_o_ methods because theire NSE values in each month and each sub-region were generally smaller than 0. Among the left 5 methods, similar to the *RE* values, the *NSE* of FAO24 and KPM methods were better, followed by the MHS_2 method, also indicating MHS_2’s better performance for the temporal variations of monthly *ET*_o_ in the methods with less climatic data demand.

NSE of the monthly and annual *ET*_o,i_ using the selected 10 methods for EMC are presented in Table 5. Except *ET*_o,7_ which was estimated by the combination-based FAO24 method, there were 0, 0, 1, 2, 1, 4, 3, 0 and 0 months fell into the ranges of 0 and 1 for NSE values of *ET*_o,1_, *ET*_o,2_, *ET*_o,3_, *ET*_o,4_, *ET*_o,5_, *ET*_o,6_, *ET*_o,8_, *ET*_o,9_ and *ET*_o,10_, respectively. This indicated a better performance of the MHS_2 method estimated by Berti *et al*.^68^ in the non-combination type. For the whole year, only *NSE*s of the FAO24 and MHS_2 methods were larger than 0, which showed the feasibility of the two methods. But for a best alternative, the combination based FAO24 was not suitable.

**Table 5:** Nash-Sutcliffe efficiency coefficients of *ET*_o,i_ in different sub-regions and EMC.

*ET*_o,i_	*ET*_o,1_	*ET*_o,2_	*ET*_o,3_	*ET*_o,4_	*ET*_o,5_	*ET*_o,6_	*ET*_o,7_	*ET*_o,8_	*ET*_o,9_	*ET*_o,10_
Month/Year										
Jan	−4.39	−9.27	−7.18	0.10	−0.93	−2.87	0.76	−0.62	−96.0	−55.7
Feb	−2.08	−3.73	−4.20	−1.49	−0.54	0.13	0.87	−0.06	−80.9	−39.0
Mar	−10.2	−4.50	−2.78	−6.47	−3.40	0.44	0.93	0.21	−92.2	−35.4
Apr	−52.1	−14.0	−4.26	−16.7	−10.5	−0.34	0.81	0.31	−136	−57.7
May	−88.5	−20.9	−0.73	−23.0	−10.3	−1.12	0.83	−0.09	−146	−84.8
Jun	−103	−22.4	0.46	−37.0	−6.88	−4.11	0.95	−10.5	−162	−119
Jul	−113	−21.4	−4.76	−33.5	−0.26	−2.03	0.80	−16.3	−207	−164
Aug	−104	−20.5	−5.52	−21.0	0.07	0.20	0.74	−11.8	−209	−171
Sep	−124	−35.9	−0.81	−16.9	−2.49	0.66	0.86	−10.1	−280	−222
Oct	−72.3	−31.7	−1.97	−2.45	−0.37	−2.21	0.86	−0.01	−233	−160
Nov	−25.0	−19.2	−6.60	0.21	−1.10	−5.08	0.71	0.44	−151	−94.8
Dec	−12.9	−16.8	−9.07	−0.61	−2.50	−6.55	0.66	−0.74	−124	−78.9
Year	−245	−76.8	−0.55	−52.4	−7.39	0.12	0.91	−5.93	−678	−439

Therefore, through a comprehensive comparison of spatiotemporal variations from the ten selected methods by relative error, standard deviation and Nash-Sutcliffe efficiency coefficient, the MHS_2 method was preliminary selected as a better one for an alternative of *ET*_o,s_ with its equation simplicity, least data demand and better performance.

### Scatter plots of monthly *ET*_o,i_ vs. *ET*_o,s_

**Figure 9:**
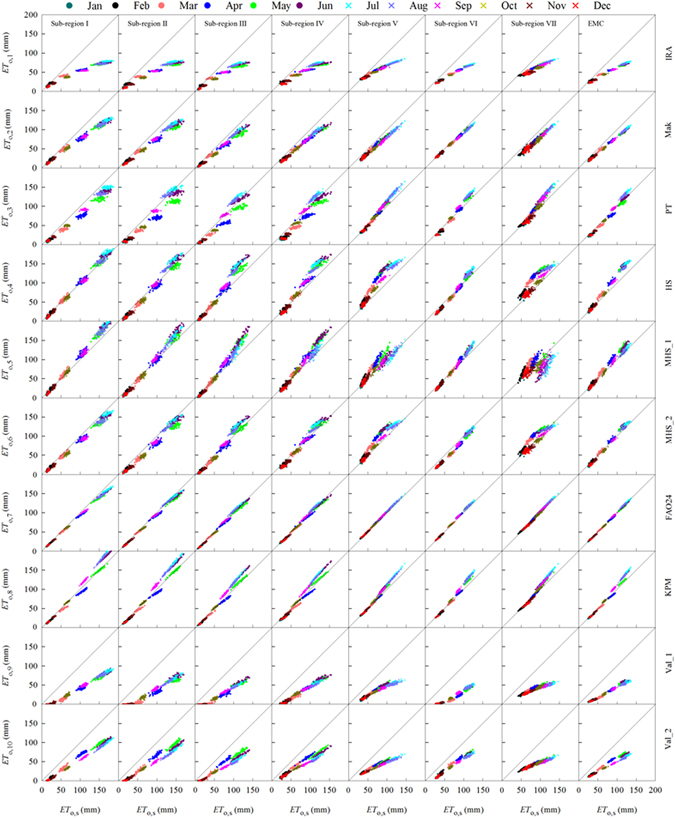
Comparisons of *ET*_o,i_ and *ET*_o,s_ in each month and sub-region.

Although the spatiotemporal distribution of multi-year mean *ET*_o,i_ were analyzed and the performances of all the methods were compared for each sub-region, direct comparison between monthly *ET*_o,i_ and *ET*_o,s_ are still necessary, in order that if the required full climatic data for estimating *ET*_o,s_ are lacking, an relatively accurate alternative method could be selected out from the 10 candidate methods using less weather data. The scatter plots of monthly *ET*_o,i_ with *ET*_o,s_ for different sub-regions and EMC are illustrated in Fig. 9. In general, *ET*_o,i_ deviated more with *ET*_o,s_ in July, but less for December and January. In all of 7 sub-regions and EMC, *ET*_o,1_, *ET*_o,2_, *ET*_o,9_ and *ET*_o,10_ were smaller but *ET*_o,4_ and *ET*_o,8_ were larger than *ET*_o,s_. *ET*_o,3_, *ET*_o,5_ *ET*_o,6_ and *ET*_o,7_ were not consistently larger or smaller than *ET*_o,s_ in different sub-regions. *ET*_o,6_ and *ET*_o,7_ were close to *ET*_o,s_, of which, data points of *ET*_o,7_ concentrated to the 1:1 lines the most. Of the 10 *ET*_o,i_, *ET*_o,9_ deviated the greatest from *ET*_o,s_, followed by *ET*_o,10_ which showed large scattered distances with the 1:1 lines. From visual comparison, *ET*_o,2_, *ET*_o,3_, *ET*_o,4_, *ET*_o,5_, *ET*_o,6_, *ET*_o,7_ and *ET*_o,8_ tended to concentrated to a striating in spite of their deviations from 1:1 line and had good linear correlations with *ET*_o,s_ in all 7 sub-regions and EMC.

By comprehensive comparisons using *RE*, standard deviations, NSE and scatter plots, although the two equations proposed by Valiantzas^14^ are relatively new, both had worse performance than the other methods in different sub-regions and EMC. The two combination type equations Doorenbos-Pruitt^4^ and Wright^69^ performed generally well, but had high weather data requirments. The equations proposed by Irmak *et al*. (2003), Makkink^66^, Priestley-Taylor^23^, Hargreaves-Samani^16^ and Droogers-Allen^67^ were all simple equations with less data requirements but didn’t perform very well. The Berti *et al*.^68^ equation (MHS_2) was a newly proposed temperature-based equation based on modified Hargreaves-Samani. The MHS_2 equation met the least data demand and had general best performance in either the empirical-based, radiation-based, temperature-based or the simplified FAO56-PM equations.

### Validation of a best alternative equation for *ET*_o,s_

For most sub-regions and EMC, there were good linear correlations between monthly *ET*_o,i_ and *ET*_o,s_. The linear equation is written as:

12 $$E{T}_{{\rm{o}},{\rm{i}}}=a\,E{T}_{{\rm{o}},{\rm{s}}}+b\,{\rm{or}}\,E{T}_{{\rm{o}},{\rm{s}}}=\frac{E{T}_{o,i}-b}{a}$$

where *a* and *b* are fitted coefficients.

Values of *a*, *b* and coefficient of determination (*R* ^2^) for various *ET*_o,i_ and 7 different sub-regions as well as EMC are given in Table 6. *R* ^2^ values for *ET*_o,1_, *ET*_o,2_, *ET*_o,3_, *ET*_o,4_, *ET*_o,6_, *ET*_o,7_, *ET*_o,8_, *ET*_o,9_ and *ET*_o,10_ were larger than 0.85 for each sub-region and EMC. Of these, the estimation of *ET*_o,1_, *ET*_o,2_, *ET*_o,3_, *ET*_o,7_, *ET*_o,8_, *ET*_o,9_ and *ET*_o,10_ utilized 5, 4, 5, 6, 6, 4 and 4 climatic variables among *T*_min_, *T*_ave_, *T*_max_, *RH*, *U*_2_, *n* and *P*, respectively; whereas *ET*_o,4_ and *ET*_o,6_ used only 3 (i.e., *T*_min_, *T*_ave_ and *T*_max_) with much simpler computation procedures.

**Table 6:** The fitted *a*, *b* and *R* ^2^ values for correlating *ET*_o,i_ with *ET*_o,s_ in different sub-regions using Equation 12.

*ET*_o,i_		*ET*_o,1_	*ET*_o,2_	*ET*_o,3_	*ET*_o,4_	*ET*_o,5_	*ET*_o,6_	*ET*_o,7_	*ET*_o,8_	*ET*_o,9_	*ET*_o,10_
Sub-region/Parameter											
I	a	0.40	0.71	0.87	1.06	1.17	0.93	0.93	1.15	0.56	0.66
	b	10.5	4.18	−6.56	−5.35	−7.32	−4.73	0.17	−9.02	−12.2	−8.19
	R^2^	0.97	0.99	0.98	0.99	0.99	**0**.**99**	0.99	0.98	0.98	0.98
II	a	0.47	0.78	0.97	1.14	1.19	1.00	0.97	1.16	0.54	0.67
	b	6.33	−0.57	−10.9	−12.7	−13.6	−11.1	−1.50	−8.46	−13.8	−10.7
	R^2^	0.96	0.98	0.94	0.98	0.99	**0**.**98**	0.99	0.98	0.96	0.97
III	a	0.58	0.85	1.08	1.36	1.31	1.19	1.02	1.16	0.54	0.62
	b	4.31	−2.87	−9.79	−12.6	−11.7	−11.0	−1.91	−5.81	−9.72	−7.47
	R^2^	0.96	0.98	0.93	0.98	0.98	**0**.**98**	0.99	0.98	0.96	0.95
IV	a	0.53	0.83	1.13	1.34	1.26	1.17	1.02	1.19	0.62	0.60
	b	8.32	−2.81	−16.0	−13.9	−11.6	−12.2	−2.84	−11.4	−14.8	−4.06
	R^2^	0.95	0.98	0.94	0.98	0.97	**0**.**98**	0.99	0.98	0.98	0.95
V	a	0.51	0.86	1.23	1.32	0.98	1.15	1.03	1.20	0.51	0.46
	b	16.1	−4.51	−10.5	0.37	8.35	0.28	−0.50	−9.50	−2.88	4.37
	R^2^	0.97	0.98	0.99	0.93	0.90	**0**.**93**	0.99	0.99	0.94	0.95
VI	a	0.54	0.90	1.25	1.24	1.23	1.09	1.07	1.23	0.53	0.71
	b	7.00	−2.32	−15.0	−18.6	−15.3	−16.2	−2.44	−11.2	−18.3	−10.8
	R^2^	0.98	0.99	0.98	0.98	0.99	**0**.**98**	0.99	0.99	0.95	0.97
VII	a	0.50	0.88	1.34	1.08	0.39	0.93	1.06	1.27	0.37	0.38
	b	16.3	−6.65	−22.3	11.7	45.2	10.4	−3.68	−18.9	7.49	10.4
	R^2^	0.94	0.97	0.97	0.85	0.31	**0**.**85**	0.99	0.98	0.89	0.94
EMC	a	0.52	0.83	1.13	1.28	1.12	1.12	1.02	1.20	0.55	0.58
	b	10.2	−2.02	−11.7	−9.17	−4.26	−8.04	−1.50	−10.5	−9.81	−3.69
	R^2^	0.98	0.99	0.98	0.99	0.98	**0**.**99**	0.99	0.98	0.98	0.96

Because temperature data are easier with less cost to observe, and *ET*_o,6_ estimated by the MHS_2 method was not only simpler, highly correlated with *ET*_o,s_ in each month and most sub-regions, but also had generally good similarity in spatiotemporal distribution with *ET*_o,s_. Considering both good performance and the correlation with *ET*_o,s_, the MHS_2 method was generally good for substituting *ET*_o,s_. Therefore, *ET*_o,6_ was finally selected as the best alternative for estimating *ET*_o,s_ in EMC. The calibrated *a* values were 0.93, 1.00, 1.19, 1.17, 1.15, 1.09, 0.93 and 1.12, and *b* values were −4.73, −11.1, −11.0, −12.2, 0.28, −16.2, 10.4 and −8.04 for sub-regions I, II, III, IV, V, VI, VII and EMC, respectively.

The best alternative MHS_2 could then be widely applied in China for *ET*_o_ estimation when only temperature data are available. Because there were still deviations in the MHS_2 method, the linear equation correlated for *ET*_o,6_ and *ET*_o,s_ using Equation 12 could be rewritten as follows:

13 $$E{T}_{{\rm{o}},{\rm{s}}}=A\,E{T}_{{\rm{o}},6}+B$$

where *A* and *B* are numerically equal to 1/*a* and −*b*/*a*, respectively. Equation 13 is also a calibration between *ET*_o,6_ and *ET*_o,s_.

**Figure 10:**
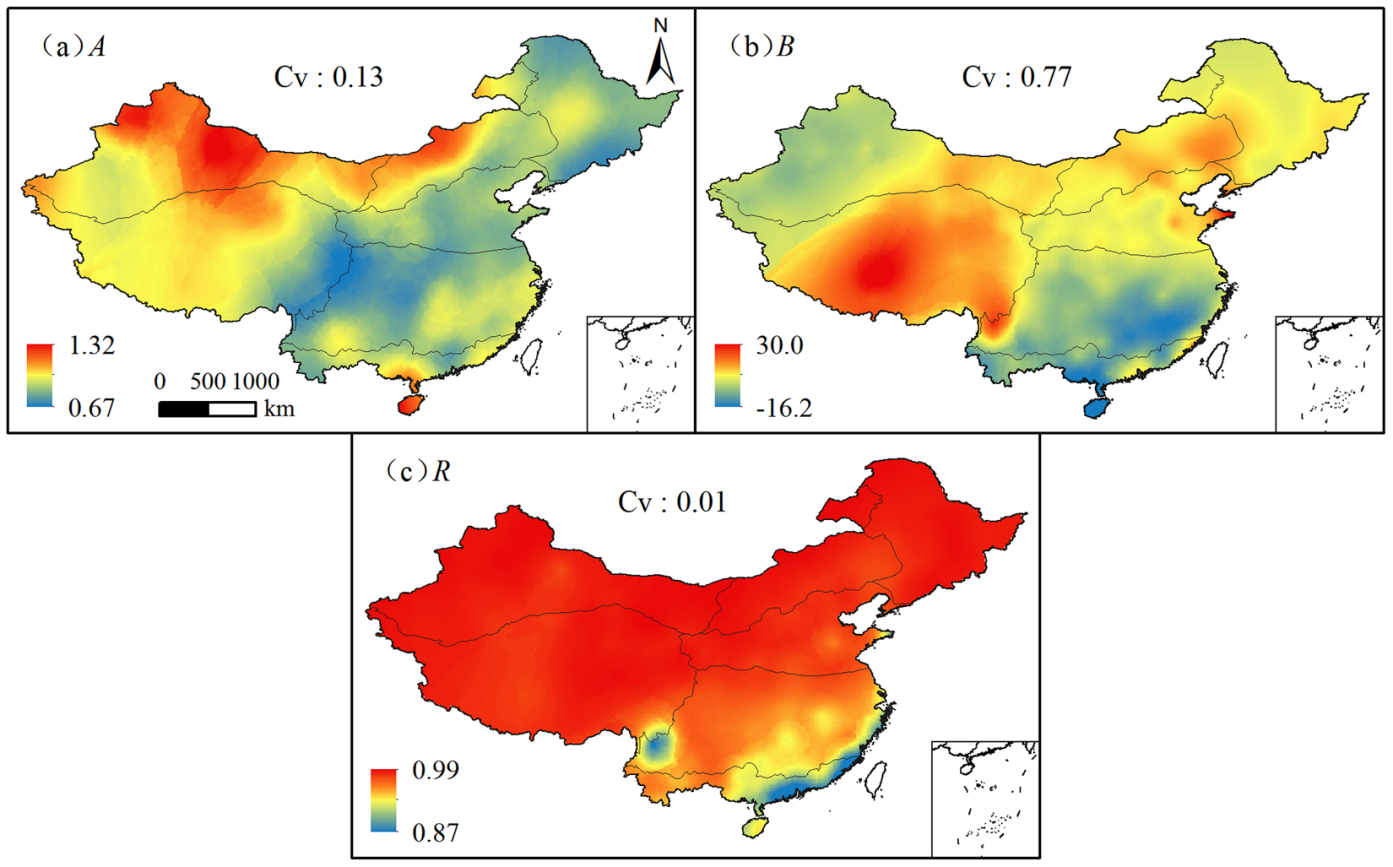
Spatial distributions of the parameters *A*, *B* and *R* in equation 13 in EMC. (ArcGIS 10.2, http://map.baidu.com, Lingling Peng).

For easier application of Eq. 13, values *A* and *B* for the 552 sites in China were validated. Figure 10 indicates the spatial distribution of *A*, *B*, and correlation coefficient (*R*). Values of *A* decreased from 1.32 to 0.67 from northwest to southwest and eastern China. *B* values were the largest in sub-region VI, followed by sub-regions II, III, IV, I, V, and VII, respectively. Values of *R* ranged between 0.87 and 0.99, were larger than 0.95 in most of China, especially in north China. The general high *R* values confirmed the applicability of the best alternative MHS_2 method in China after accurate calibration.

## Discussion

Under the global climate change, decreasing trends in *ET*_o_ have been observed in different parts of the world^32, 76, 77^, including China^78^ and most parts of China, e.g., the Haihe River basin^79^, the Huang-Huai-Hai Plain^80^, the northwest China including Xinjiang Uywer Autonomos Region^81^, southeast China, the Yangtze river basin^64^, etc. The increasing trends were found at most sites of the Qinghai-Tibetan Plateau^34^. The trends were also bi-directional in China. This study revealed that annual *ET*_o,s_ for 61.4% of the study sites had decreasing trends, of them, 9% of the trends were significant. Our research agreed with the former research in the general decreasing trends of *ET*_o,s_ for EMC, but in the meantime, there were also differences between this research and the previous.. The differences were caused by the changes in the study period, the data source, the *ET*_o,s_ estimation methods, the site number applied, and the research aims. For example, Wang *et al*.^51^ also applied 4189-grid data during 1961–2013 in EMC to estimate *ET*_o_ and identified the contribution of climatic variables to *ET*_o_ variability. They revealed that annual *ET*_o_ decreased with a mean rate of 6.84 mm/decade, and the sites with significant increase trends mainly distributed in the Qinghai-Tibetan Plateau. This research also reported general increasing trends in the same region, i.e. sub-region VI.

The most precise *ET*_o_ estimation method varied for different regions. The frequently-used methods are the FAO56-PM, HS, and pan measurement etc., these methods have been applied to partial of China or EMC^58, 78, 82^. Xu *et al*.^82^ applied 5 meteorological stations during 1999–2007 in arid-zone of China (i.e., sub-region I, VI of this research) and selected the HS method as the best alternative of *ET*_o,s_. This research selected the MHS_2 as a best alternative of *ET*_o,s_ for different sub-regions and EMC, because it not only had a general high accuracy but also used only temperature data which were easy to observe or collected, even for the sites where the other climatic data were lacking. Moreover, this research also provided the spatial distributions of the calibrated parameters of the MHS_2 method as the best alternative of *ET*_o,s_ for different sub-regions and EMC, which were very useful for researchers to apply the calibrated MHS_2 method in China.

The MHS_2 method overestimated *ET*_o_ in the sub-regions V and VII in the high temperature section of EMC (Fig. 7f). *RE* reached 20% especially in March, April, May and June (Figs 8e,g and 9). Both sub-regions are humid and sub-humid climatic zones of EMC. This reflected the disadvantages of MHS_2 which only applied temperature data for estimating *ET*_o_. When temperature is high, *ET*_o,6_ obtained with the MHS_2 method could be high but *ET*_o,s_ may not be as high as it considering also wind speed, relative humidity and sunshine hour. Under the overestimation conditions, the relationship between *ET*_o,6_ and *ET*_o,s_ should be re-calibrated for March, April, May and June. The re-calibrated parameters *A*, *B* and *R* ^2^ in March, April, May and June for the two sub-regions are presented in Table 7.

**Table 7:** The re-calibrated parameters *A*, *B* and *R* ^2^ in March, April, May and June for the V and VII sub-regions using Equation 13.

Month	March			April			May			June		
Sub-region	A	B	R^2^	A	B	R^2^	A	B	R^2^	A	B	R^2^
V	0.99	−14.4	0.81	1.02	−24.7	0.88	1.18	−47.5	0.91	1.47	−82.2	0.84
VII	1.23	−38.4	0.75	1.56	−78.1	0.78	2.00	−133.4	0.82	2.13	−136.8	0.89

## Conclusions

Based on monthly climatic data collected from 552 stations during 1961–2013 across different sub-regions of China, a comprehensive comparison between *ET*_o,i_ (estimated by the IRA, Mak, PT, HS, MHS_1, MHS_2, FAO24, KPM, Val_1 and Val_2 methods) and *ET*_o,s_ estimated by the FAO56- PM method has been conducted in 7 sub-regions and EMC. 339 and 213 sites had decrease and increase trends in annual *ET*_o,s_, indicating a general decrease trend in annual *ET*_o,s_. For the spatial distribution, values of multi-year mean monthly *ET*_o,s_ in western China (high elevations) were larger than in eastern China (low elevations). The step by step comparison of spatiotemporal distribution, *RE*, standard deviations, NSE and scatter plots between *ET*_o,i_ and *ET*_o,s_ either for monthly and annual timescales or different sub-regions and ECM consistently showed the general high accuracy of *ET*_o,6_ estimated by the MHS_2 method proposed by Berti *et al*.^68^. The MHS_2 method utilized only temperature data, was simple in computation procedure when compared to the other 9 *ET*_o_ estimation methods, and was highly correlated with *ET*_o,s_. It was a best alternative for *ET*_o,s_ when climatic data were lacking. After accurate validation for the MHS_2 method using equation 13, the calibrated parameters of *A* and *B* for each site, sub-region and EMC were obtained. This research is an important contribution to *ET*_o_ estimation method in China when the high requirements of climatic data could not be met.

## Electronic supplementary material

Supplementary Information
