# Supplementary material for: The best alternative for estimating reference crop evapotranspiration in different sub-regions of mainland China
Source: Sci Rep. 2017 Jul 14;7:5458. doi: 10.1038/s41598-017-05660-y (PMC5511263; doi:10.1038/s41598-017-05660-y)
Supplement: Supplementary file 1 — Supplementary Information [file 41598_2017_5660_MOESM1_ESM.pdf]

The best alternative for estimating reference crop evapotranspiration in different sub-regions of mainland China

Lingling Peng[1, 2,] Yi Li[1, 2,*], Hao Feng[2, 3]

1 College of Water Resources and Architecture Engineering, Northwest Agriculture and Forestry University, Yangling, Shaanxi, 712100, China.

2 Institute of Water-Saving Agriculture in Arid Areas of China, Northwest A&F University, Yangling 712100, China.

3 Institute of Soil and Water Conservation, Chinese Academy of Sciences and Ministry of Water Resources, Yangling 712100, China.

* Correspondence: Yi Li, Email: liyikitty@126.com

Supplementary Figures

Figure S1 The comparison of monthly $ET_{o,i}$ for the total 16 selected methods.

Figure S2 Spatial distribution of multi-year mean monthly standard deviations of $ET_{o,i}$ in EMC. (ArcGIS 10.2, http://map.baidu.com, Lingling Peng)

Figure S3 The monthly temporal variations of multi-year mean standard deviations of $ET_{o,i}$ in different sub-regions and EMC.

Figure S4 Spatial distribution of multi-year mean monthly Nash-Sutcliffe efficiency coefficients (*NSE*s) of $ET_{o,i}$ in EMC. (ArcGIS 10.2, http://map.baidu.com, Lingling Peng)

Figure S5 The temporal variations of multi-year mean monthly *NSE*s for $ET_{o,i}$ in different sub-regions and EMC.

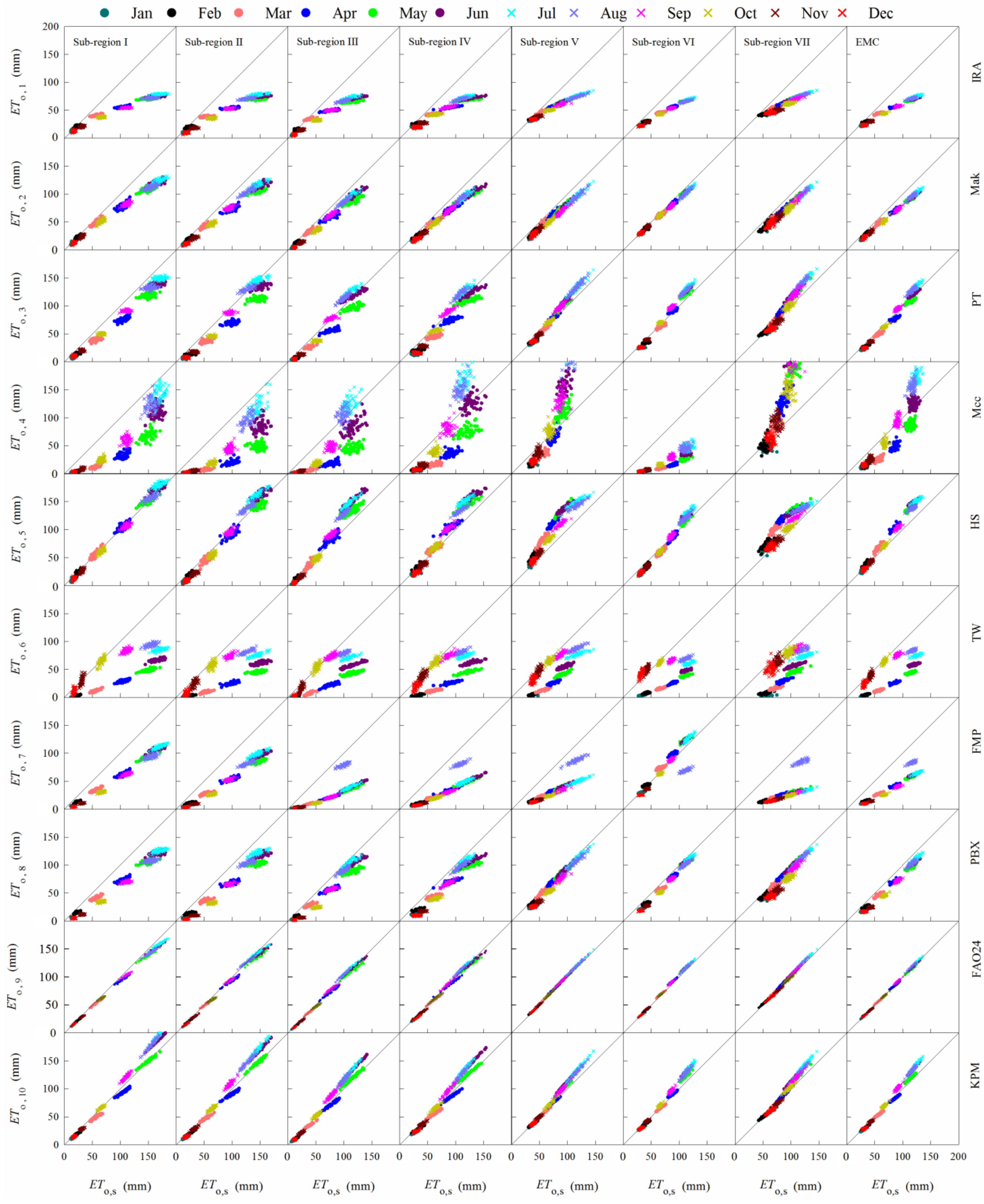

Figure S1 The comparison of monthly $ET_{\mathrm{o,i}}$ for the total 16 selected methods.

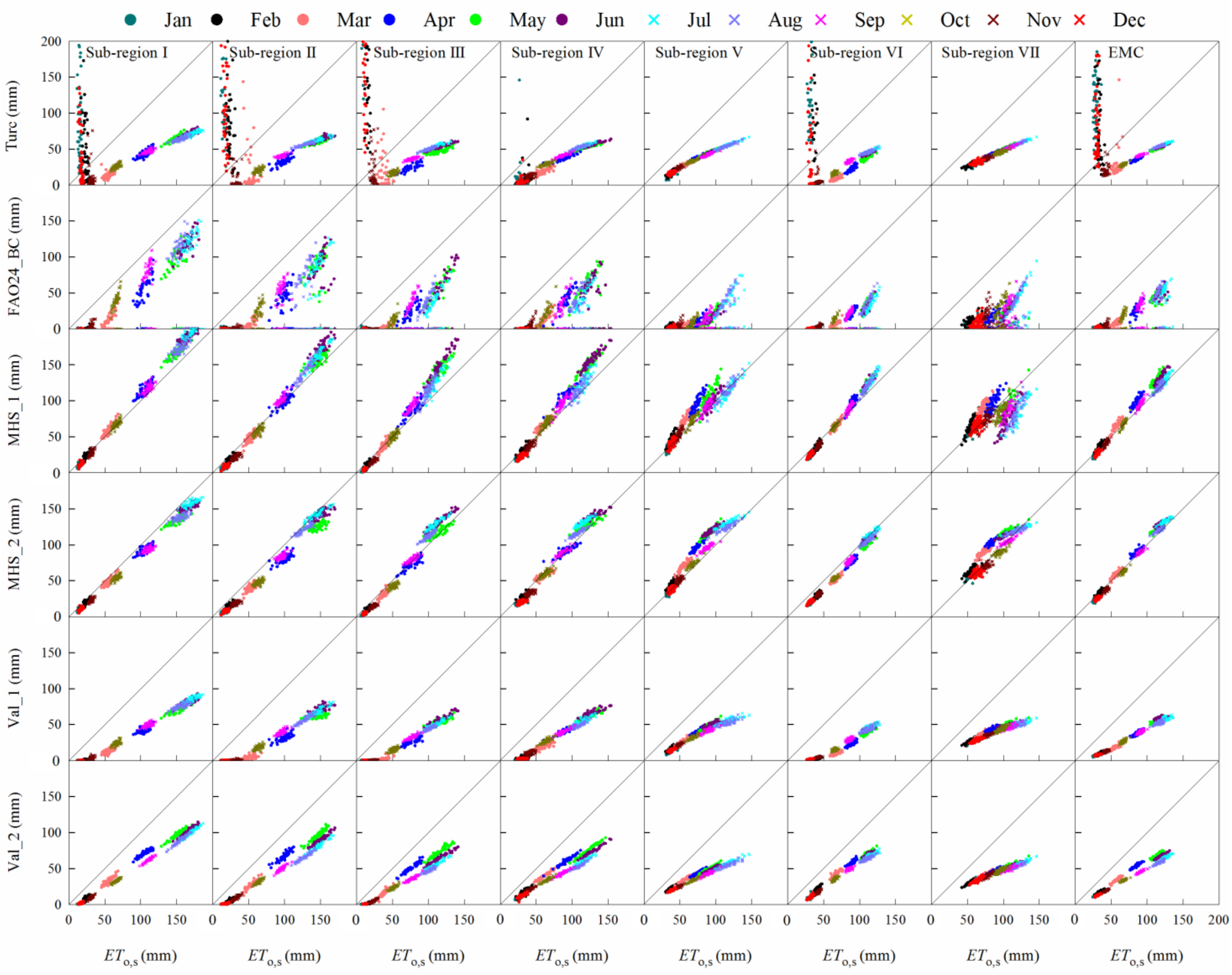

Continued Figure S1 The comparison of monthly $ET_{o,i}$ for the total 16 selected methods.

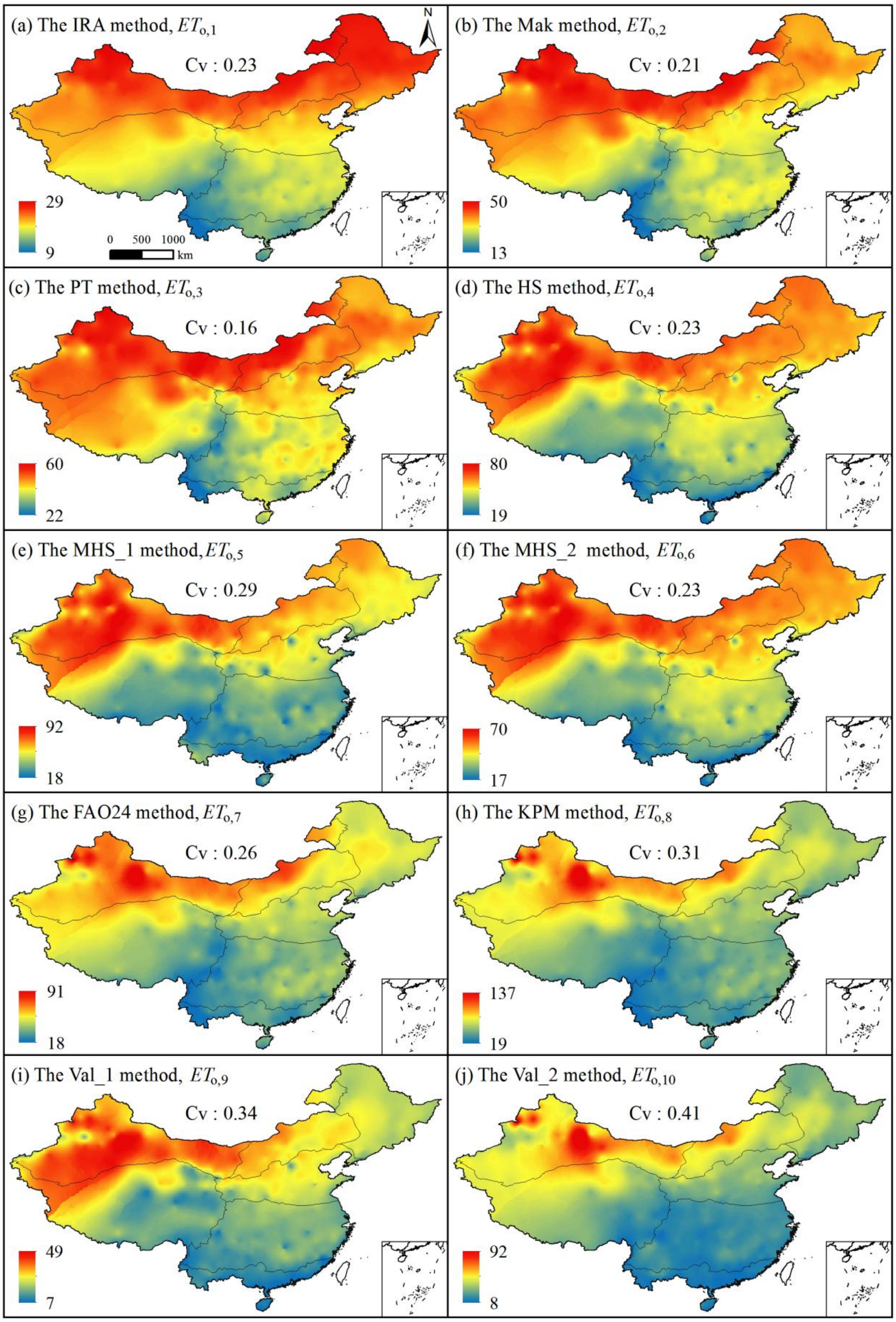

Figure S2 Spatial distribution of multi-year mean monthly standard deviations of $ET_{o,i}$ in EMC. (ArcGIS 10.2, http://map.baidu.com, Lingling Peng)

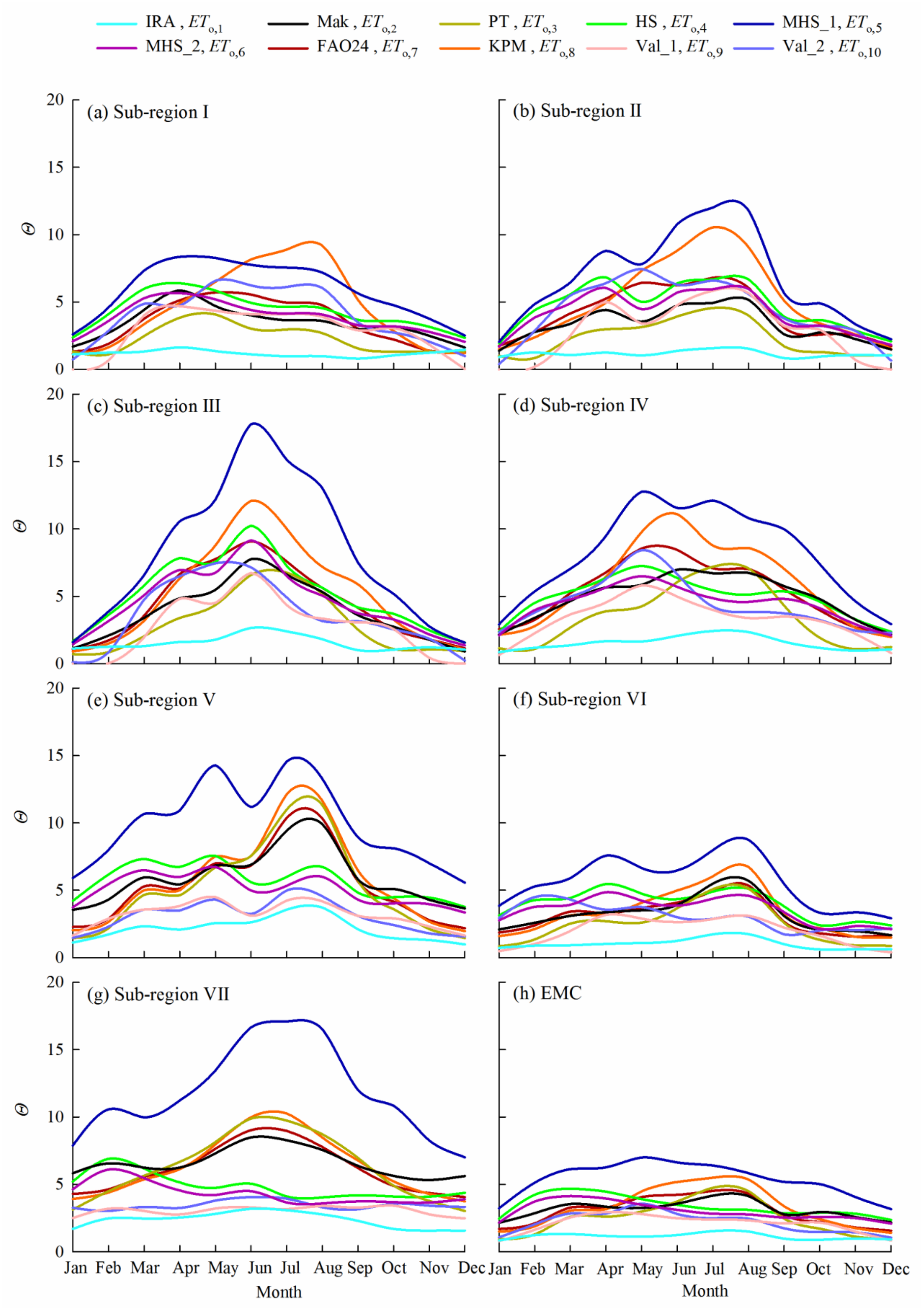

Figure S3 The monthly temporal variations of multi-year mean standard deviations of $ET_{o,i}$ in different sub-regions and EMC.

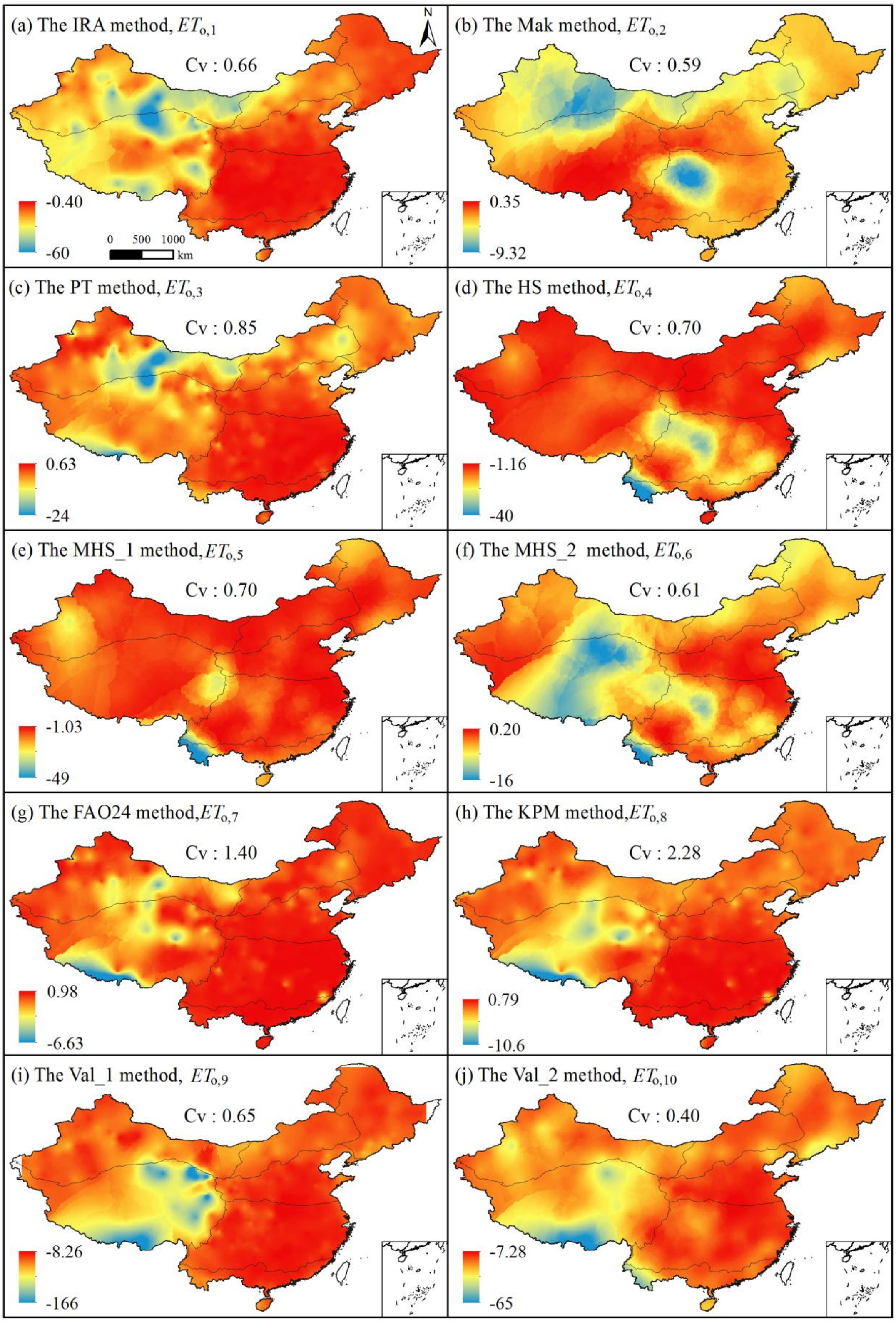

Figure S4 Spatial distribution of multi-year mean monthly Nash-Sutcliffe efficiency coefficients (*NSE*s) of $ET_{o,i}$ in EMC. (ArcGIS 10.2, http://map.baidu.com, Lingling Peng)

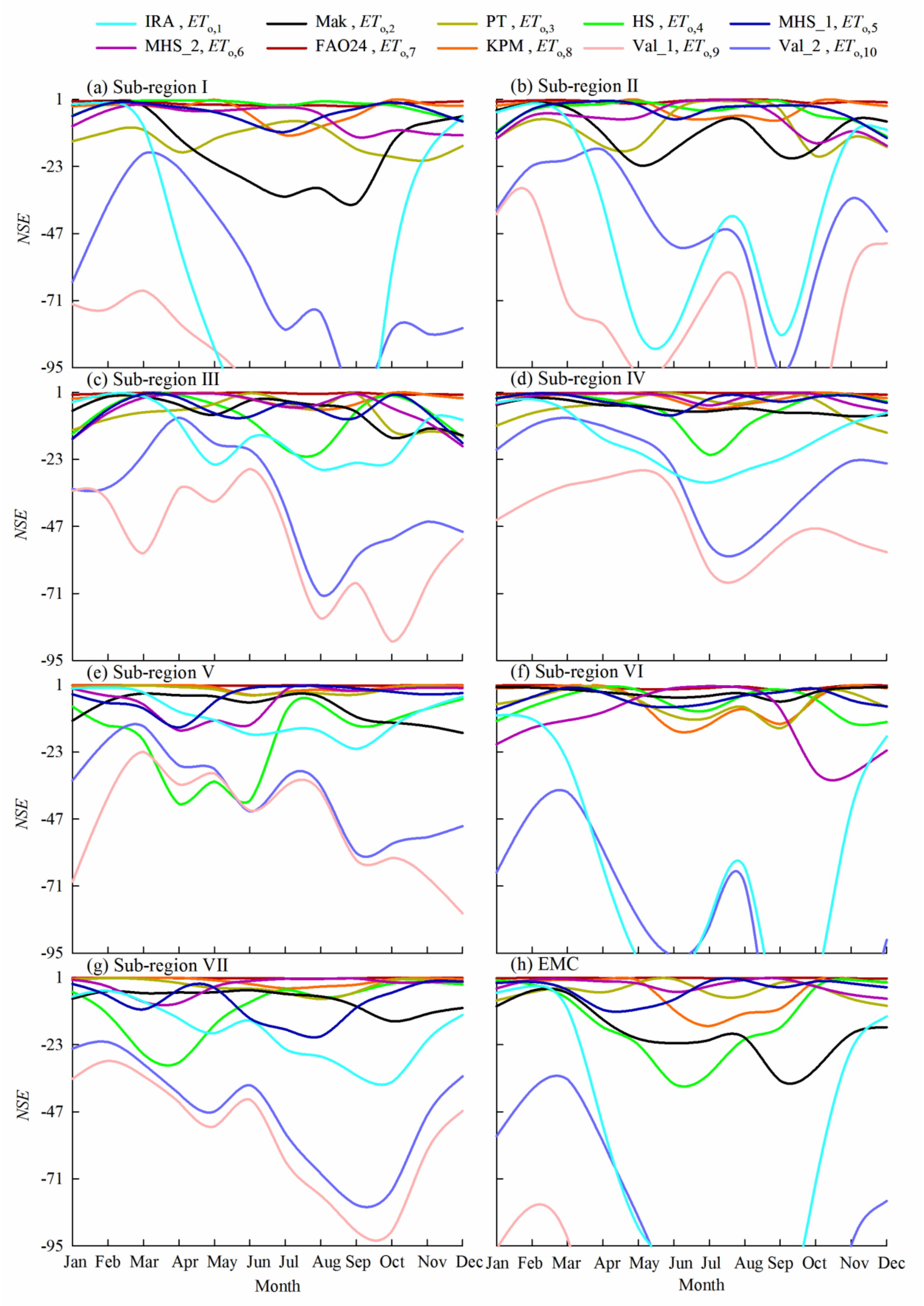

Figure S5 The temporal variations of multi-year mean monthly *NSE*s for $ET_{o,i}$ in different sub-regions and EMC.
